# Antisense oligonucleotide targeting TARDBP-EGFR splicing axis inhibits progression of oral squamous cell carcinoma through ABCA1-regulated cholesterol efflux

**DOI:** 10.1038/s41368-025-00402-7

**Published:** 2026-01-16

**Authors:** Nan Ni, Moxu Wang, Zhiran Yuan, Leqi Zhang, Jilin Cai, Qingqing Du, Pengcheng Li, Chang Gao, Hanwen Zhang, Yuancheng Li, Hua Yuan

**Affiliations:** 1 Jiangsu Key Laboratory of Oral Diseases, Nanjing Medical University, Nanjing, China; 2 Department of Oral and Maxillofacial Surgery, Affiliated Hospital of Stomatology, Nanjing Medical University, Nanjing, China; 3 Department of Oral and Dental Implantology, Affiliated Hospital of Stomatology, Nanjing Medical University, Nanjing, China; 4 Central Research Laboratory, Jiangsu Key Laboratory of Molecular Biology for Skin Diseases and STIs, Institute of Dermatology, Chinese Academy of Medicine Sciences and Peking Union Medical College, Nanjing, China; 5 Jiangsu Province Engineering Research Center of Stomatological Translational Medicine, Nanjing, China; 6 Jiangsu Key Lab of Cancer Biomarkers, Prevention and Treatment, Collaborative Innovation Center for Cancer Personalized Medicine, Nanjing Medical University, Nanjing, China

**Keywords:** Cancer epidemiology, Oral cancer

## Abstract

Splice quantitative trait loci (sQTL) serve as another critical link between genetic variations and human diseases, besides expression quantitative trait loci (eQTL). Their role in oral squamous cell carcinoma (OSCC) development remains unexplored. We collected surgically resected cancer and adjacent normal epithelial tissue samples from 67 OSCC cases, and extracted RNA for sequencing after quality control. A genome-wide sQTL analysis was performed using the RNA sequencing data from 67 normal oral epithelial tissue samples. We included peripheral blood DNA samples from 1044 patients with OSCC and 3199 healthy controls to conduct a genome-wide association study. Systematic screening of sQTLs associated with OSCC risk identified a sQTL variant—the rs737540-T allele—independent of eQTLs, significantly associated with an increased risk of OSCC (OR = 1.2, *P* = 6.84 × 10^−4^). The rs737540-T allele reduced skipping of EGFR alternative exon 4 by enhancing TAR DNA binding protein (TARDBP) binding to the RNA sequence, leading to increased expression of the longer isoform (*EGFR-001*) and reduced expression of the truncated isoform (*EGFR-004*). Compared with *EGFR-004*, *EGFR-001* promoted OSCC cell proliferation by reducing ATP-binding cassette subfamily A member 1 (ABCA1) ubiquitination through lower EGFR phosphorylation. ABCA1 was demonstrated to increase the cholesterol content of the plasma membrane via cholesterol efflux, thus affecting membrane fluidity and vimentin-mediated epithelial–mesenchymal transition. An antisense oligonucleotide targeting rs737540 significantly inhibited OSCC proliferation and reversed membrane cholesterol-induced resistance. This study provides novel insights into how genetic variants regulating alternative splicing contribute to OSCC risk and identifies potential therapeutic targets.

## Introduction

Oral squamous cell carcinoma (OSCC) is an epithelium-derived malignant tumor that occurs in the tongue, gums, cheeks, palate, and floor of the mouth. Its prognosis remains unsatisfactory despite recent treatment advances and improved survival rates.^1^ It is crucial to deepen our understanding of the pathogenesis of OSCC to develop personalized treatment strategies for high-risk individuals.

Precursor messenger RNA (pre-mRNA) can generate multiple transcripts of a single gene through splicing. This is an important post-transcriptional regulatory mechanism to maintain gene and protein diversity in the body, present in over 90% of genes in the human genome.^2,3^ Tumors exhibit higher alternative splicing events than adjacent normal tissues, which can promote proliferation, metastasis, chemotherapy resistance, and immune evasion.^4,5^ Splicing quantitative trait locus (sQTL) analysis has emerged as an identification method for genetic variants associated with alternative splicing. Thus, integrating sQTL with genome-wide association studies (GWAS) can help identify more actual disease-causing genetic variants. The Genotype-Tissue Expression (GTEx) project reported that 37% of sQTLs were disease- or trait-related loci according to GWAS.^6^ Moreover, sQTL studies regarding colorectal cancer and lung cancer have linked splicing-related variants to tumor risk.^7,8^ However, sQTLs exhibit tissue specificity, and their effect on the risk of OSCC remains largely unexplored.^6^ Considering this, there is a need to systematically identify OSCC-associated splicing variants and elucidate their biological mechanisms in regulating specific target genes and tumorigenesis. This should help deepen our understanding of the etiology of OSCC, and the new susceptibility loci discovered will help guide the screening of high-risk populations.

Herein, we identified *EGFR* as a target gene whose splicing isoforms are regulated by rs737540 variant. *EGFR* is overexpressed in over 90% of head and neck squamous cell carcinomas.^9^ Wild-type *EGFR* signal transduction contributes to apoptosis evasion, proliferation, angiogenesis, and metastasis.^10^ Cetuximab, an FDA-approved *EGFR*-targeted therapy for advanced OSCC,^11^ is associated with limitations due to resistance to it developing via alternative pathway activation.^12^ Additionally, mutations originating from alternative splicing of *EGFR* have been demonstrated to affect patient responsiveness to drug therapy. For example, *EGFR* exon 19 deletion alters patient sensitivity to tyrosine kinase inhibitors (TKIs).^13^

Abnormal alternative splicing also presents a therapeutic target. For example, tepotinib is used for non-small cell lung cancer with a MET exon 14 skipping mutation.^14^ Thus, targeting *EGFR* splicing elements to suppress aggressive and resistant isoforms is a promising strategy. Antisense oligonucleotides (ASOs), which bind specific RNA-target sequences and block splicing factors binding sterically to the pre-mRNA, offer a specific option to modulate target RNA sequences.^15^ For example, ASO-mediated exon 6 skipping was reported to decrease the abundance of MDM4 and inhibit melanoma growth.^16^ Therefore, identifying OSCC-associated sQTLs and designing corresponding ASOs holds substantial potential for precision medicine in high-risk populations.

## Results

### Identification of sQTLs associated with OSCC risk

**Fig. 1:**
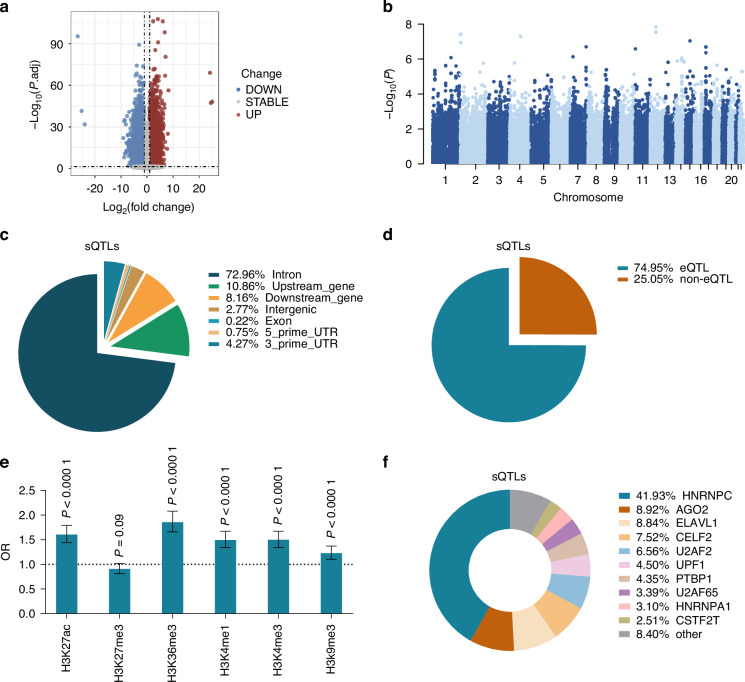
Identification of sQTLs associated with OSCC risk in normal oral epithelial tissue. **a** Differentially expressed transcripts between 81 pairs of OSCC and adjacent normal oral epithelial tissues. **b** Manhattan map of sQTLs in normal oral epithelial tissue. **c** Genome distribution of sQTLs. **d** The proportion of sQTLs with eQTL effects. **e** Comparison of different histone modifications on sequences of sQTL and non-sQTL loci. **f** The top 10 potential binding RBPs on the sequence of sQTLs

RNA-seq analysis revealed 19 649 differentially expressed transcripts corresponding to 10 027 genes, satisfying the criteria of |log_2_FC|≥1 and false discovery rate (FDR) of <0.05 (Fig. 1a). Furthermore, we identified cis-sQTLs (single nucleotide polymorphism, SNPs within ±1 kb of genes) for these 10 027 genes (Fig. 1b), yielding 1 372 sQTLs, corresponding to 244 genes (gene-level FDR < 0.1). Notably, most sQTLs were located within intronic regions (Fig. 1c). eQTL analysis of sQTL-associated variants and their target genes revealed that only 25.05% lacked a significant eQTL effect (Fig. 1d). sQTLs were significantly enriched in histone modification regions (excluding H3K27me3) compared with non-sQTLs (Fig. 1e). Moreover, analysis of RNA-binding protein binding sites on sQTL sequences indicated potential roles for these proteins in regulating alternative splicing (Fig. 1f).

Following the removal of linkage disequilibrium (LD; r^2^ > 0.8), only four sQTLs were significantly associated with OSCC risk (*P* < 0.01) (Table 1). Notably, rs737540, located on chromosome 13q32.2, emerged as the most significant risk locus for OSCC in our association study (OR = 1.2, *P* = 6.84 × 10^−4^), suggesting a molecular mechanism underlying the GWAS signals.

**Table 1:** Results of association between sQTLs and risk of OSCC

Region	SNP	A1/A2	Gene	*P*_sQTL_	Beta	95%CI	*P*_GWAS_	OR	95%CI
7p11.2	rs737540	T/C	EGFR	4.53 × 10^−4^	0.59	0.40–0.79	6.84 × 10^−4^	1.2	1.08–1.33
19q13.32	rs78961563	T/C	AC006262.5	1.63 × 10^−3^	0.69	0.39–0.98	1.14 × 10^−3^	1.28	1.09–1.46
1q25.3	rs116876471	C/T	EDEM3	5.25 × 10^−3^	0.9	0.33–1.47	4.64 × 10^−3^	1.41	1.07–1.74
2q36.3	rs12694743	G/A	SLC19A3	1.63 × 10^−3^	0.78	0.40–1.16	5.23 × 10^−3^	0.82	0.70–0.93

### rs737540-T allele decreases *EGFR* exon 4 skipping mediated by TARDBP in OSCC

**Fig. 2:**
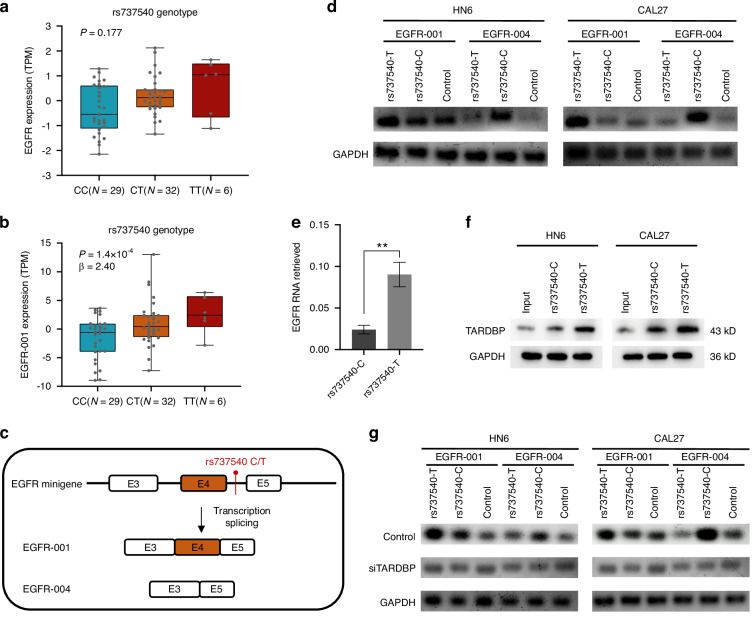
TARDBP preferentially binds to the rs737540-T allele and promotes oncogenic EGFR alternative splicing. **a** The expression of *EGFR* in patients with different rs737540 genotypes. **b** The expression of *EGFR-001* in patients with different rs737540 genotypes. **c** Differences in the sequence between *EGFR-001* and *EGFR-004*. **d** Minigene assays in HN6 and CAL27 cells were conducted to confirm the effects of the sQTL variant rs737540 on both *EGFR-001* and *EGFR-004* expression levels. **e** RIP analysis showed that TARDBP preferentially binds to the rs737540-T allele. **f** Western blotting demonstrated that EGFR binds to RNA probes via incubation of biotinylated rs737540-C or rs737540-T with protein extracts from HN6 and CAL27 cells. **g** Minigene assays in HN6 and CAL27 cells were conducted after rs737540-C or rs737540-T overexpression under TARDBP knockdown to confirm the effects of TARDBP on both *EGFR-001* and *EGFR-004* expression levels. Data are presented as the mean ± SD from three independent experiment. Statistical significance was assessed with Student’s *t*-test. **P < 0.01

rs737540 is a prominent OSCC risk locus located on chromosome 7 near *EGFR* (Supplementary Fig. 1). Association analysis revealed no eQTL effect of rs737540 on *EGFR* expression (*P*_eQTL_ = 0.177); however, a significant independent sQTL effect was observed (Fig. 2a). eQTL analysis of *EGFR* transcripts revealed that the rs737540-T allele significantly upregulated *EGFR-001* expression compared with rs737540-C allele (Fig. 2b). rs737540 is an intron variant situated between *EGFR* exons 4 and 5. Through analysis of the Ensembl database (GRCh37), we observed that *EGFR-004* lacks exon 4 compared with *EGFR-001* (Fig. 2c). This led us to hypothesize that rs737540 modulates exon 4 skipping, thus regulating *EGFR* alternative splicing and influencing splicing subtype expression.

Minigene and RT-qPCR assays confirmed that rs737540-T decreased *EGFR* exon 4 skipping compared with rs737540-C, resulting in lower *EGFR-004* levels and higher *EGFR-001* levels (Fig. 2d, Supplementary Fig. 2a). Moreover, POSTAR3 database analysis indicated stronger TARDBP binding affinity for the rs737540-T allele (Supplementary Table S1). RNA immunoprecipitation and RNA pull-down assays confirmed preferential binding of TARDBP to the rs737540-T sequence over rs737540-C (Fig. 2e, f). Knockdown of TARDBP significantly reduced *EGFR* exon 4 splicing signals in the minigene system and reversed the expression levels of *EGFR-001* and *EGFR-004* in OSCC cells (Fig. 2g, Supplementary Fig. 2b).

### ASO-mediated inhibition of TARDBP binding reduces OSCC growth

**Fig. 3:**
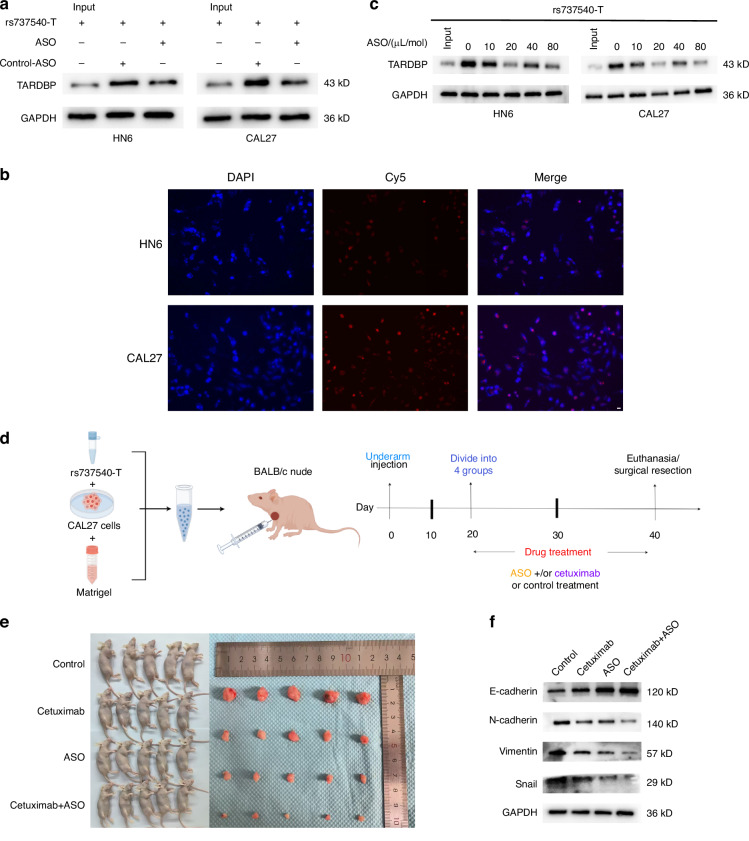
Therapeutic effects of cetuximab and/or antisense oligonucleotides in vitro and in vivo. **a** Western blotting analysis demonstrated that the ASO inhibited TARDBP binding to RNA. **b** The subcellular localization of Cy5-labeled ASO in OSCC cells was analyzed co-staining with DAPI. **c** RNA pull-down results proved the combination of rs737540-T and TARDBP at different concentrations of ASO. **d** A procedure of underarm implantation of CAL27 cells in BALB/c mice, and treated with ASO (3.375 mg/kg) and/or cetuximab (20 mg/kg) at a specified time point. **e** General images of rs737540-T overexpression of subcutaneous tumor in BALB/c mice treated with cetuximab and/or ASO. **f** Western blot assay to detect the expression levels of EMT markers in each group

To investigate rs737540 as a drug target, we designed an ASO overlapping with its location. RNA pull-down revealed that this ASO inhibited TARDBP binding to rs737540-T (Fig. 3a). The ASO localized primarily to the nucleus in OSCC cells (Fig. 3b). To determine the optimal ASO concentration, HN6 and CAL27 cells were treated with five different ASO concentrations. After 24 h, 20 µL/mol ASO maximally inhibited OSCC cell growth, whereas cetuximab exhibited a dose-dependent inhibitory effect (Supplementary Fig. 3a). Cetuximab combined with ASO was superior to either agent alone at all of the tested concentrations (Supplementary Fig. 3b). The tested combinations of five cetuximab and five ASO concentrations revealed maximal inhibition at 20 µL/mol ASO with any concentration of cetuximab (Supplementary Fig. 3c). Furthermore, RNA pull-down showed the weakest TARDBP binding at an ASO concentration of 20 µL/mol (Fig. 3c). We injected rs737540-T -overexpressing CAL27 cells subcutaneously into the axilla of nude mice and treated them with cetuximab and/or the ASO (Fig. 3d). Combination therapy most effectively inhibited tumor growth (Fig. 3e, Supplementary Fig. 4a–c) and epithelial–mesenchymal transition (EMT) (Fig. 3f, Supplementary Fig. 5a, b).

### Differential effects of *EGFR* isoforms on OSCC cells

**Fig. 4:**
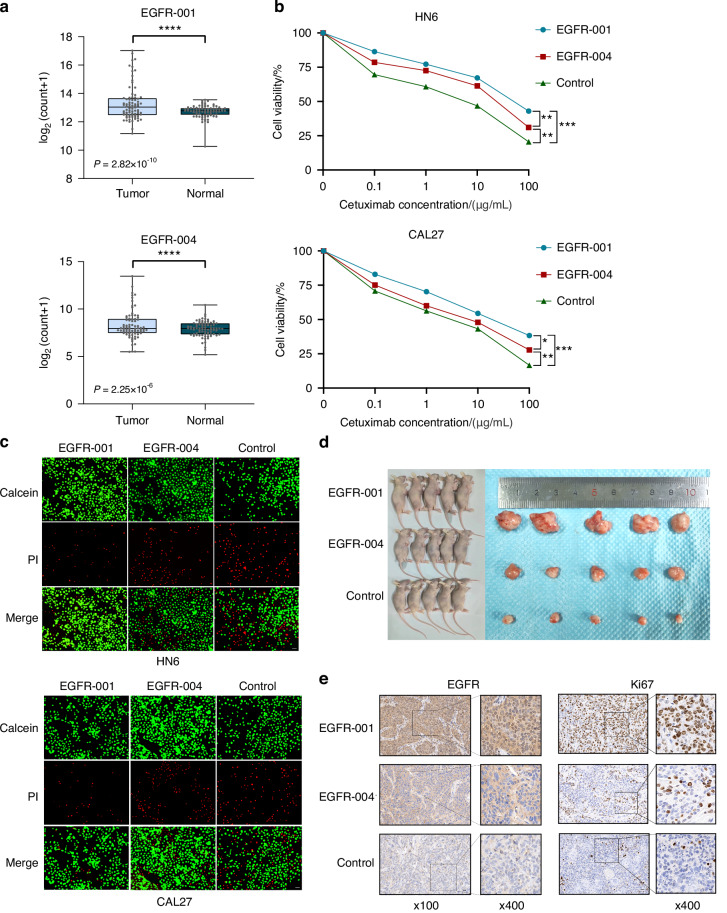
The differential effects of EGFR isoforms on tumor cells. **a** RNA-seq expression levels of *EGFR-001* and *EGFR-004* in OSCC tissues and adjacent normal tissues. **b** Viability of *EGFR-001*-overexpressing and *EGFR-004*-overexpressing cells treated with different concentrations of cetuximab. **c** Calcein/PI cell viability assays revealed the cell death rate of HN6 and CAL27 cells overexpressing *EGFR-001* and *EGFR-004*. Scale bars, 100 μm. **d** General images of subcutaneous tumors of *EGFR-001* and *EGFR-004* overexpressing CAL27 cells from a xenograft nude mouse model. **e** Immunohistochemistry (IHC) staining of EGFR and Ki67. Scale bars, 100 μm (left), 25 μm (right). Statistical significance was assessed with Student’s *t*-test. *P < 0.05, **P < 0.01, ***P < 0.001, **** *P* < 0.000 1

Distinct *EGFR-001* and *EGFR-004* expression patterns were observed in 81 paired OSCC and adjacent tissues (Fig. 4a). The levels of both transcripts were significantly higher in OSCC cells (HN6, CAL27) than in immortalized human oral keratinocytes (HOKs) (Supplementary Fig. 6). In vitro, CCK-8 assays revealed that *EGFR-001* overexpression significantly enhanced OSCC cell proliferation compared with *EGFR-004* overexpression (Supplementary Fig. 7a). Colony formation assays further confirmed the superior proliferative capacity of *EGFR-001*-overexpressing cells (Supplementary Fig. 7b). Additionally, wound healing and Transwell assays revealed that *EGFR-001* significantly increased the migration and invasiveness of OSCC cells compared with *EGFR-004* (Supplementary Fig. 7c, d). Meanwhile, *EGFR-001*-overexpressing cells exhibited greater resistance to varying concentrations of cetuximab than *EGFR-004*-overexpressing cells did (Fig. 4b), consistent with the results of calcein/PI viability assays (Fig. 4c).

In vivo tumorigenesis of nude mice revealed that *EGFR-001* overexpression significantly increased tumor volume and weight compared with *EGFR-004* overexpression (Fig. 4d, Supplementary Fig. 7e, f). In addition, EGFR and Ki67 staining revealed higher expression levels in OSCC with *EGFR-001* overexpression (Fig. 4e). We also compared the status of rs737540 tumor cells with different genotypes. The overexpression of rs737540-T significantly enhanced OSCC cell proliferation (Supplementary Fig. 8a), colony formation, migration, and invasion compared with the overexpression of rs737540-C (Supplementary Fig. 8b–d), aligning with the findings of population studies and indicating that rs737540-T elevates EGFR-001 levels and OSCC risk.

### ABCA1, a downstream signaling molecule, is differentially regulated by EGFR isoforms

**Fig. 5:**
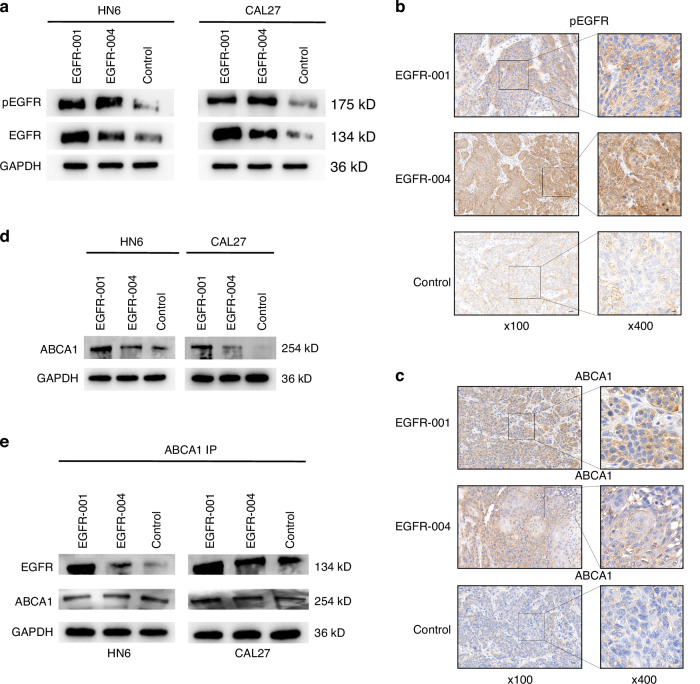
EGFR isoforms differentially inhibit cell proliferation by facilitating ABCA1 activation. **a** Western blotting analysis of the effect of *EGFR* isoform overexpression on the phosphorylation of EGFR. **b**, **c** Immunohistochemistry (IHC) staining of pEGFR and ABCA1. Scale bars, 100 μm (left), 25 μm (right). **d** The protein expression of ABCA1 in HN6 and CAL27 cells overexpressing *EGFR-001* or *EGFR-004* was detected by western blotting. **e** Co-immunoprecipitation analysis of the interaction between ABCA1 and EGFR in HN6 and CAL27 cells transfected with control, *EGFR-001*, or *EGFR-004* plasmids

*EGFR-004* overexpression significantly promoted EGFR phosphorylation in HN6 and CAL27 cells compared with the levels associated with *EGFR-001* overexpression (Fig. 5a), a pattern also observed in the tumor tissues of nude mice (Fig. 5b). As EGFR phosphorylation promotes ubiquitin-mediated ABCA1 degradation in germ cells,^17^ we hypothesized that distinct *EGFR* isoforms may exert differential effects on ABCA1. Moreover, ABCA1 protein levels were higher in EGFR-001-overexpressing nude mouse tumors and cells (Fig. 5c, d). Co-immunoprecipitation (Co-IP) confirmed interaction between EGFR and ABCA1, with *EGFR-004* overexpression resulting in less co-precipitated ABCA1 compared with *EGFR-001* (Fig. 5e).

Silencing *ABCA1* in HN6 and CAL27 cells inhibited differential proliferation (Supplementary Fig. 9a), colony formation, migration, and invasion caused by EGFR isoforms (Supplementary Fig. 9b–d).

### *EGFR* isoforms affect ABCA1 protein ubiquitination and proteasome degradation

**Fig. 6:**
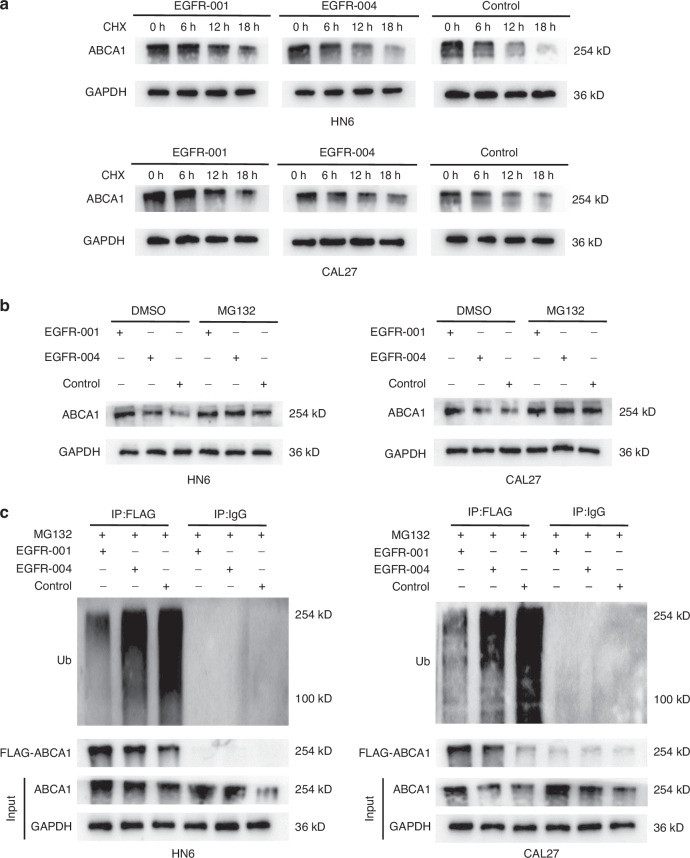
Ubiquitination levels of different EGFR isoforms. **a** Western blotting showing the ABCA1 protein in CAL27 and HN6 cells transfected with control, *EGFR-001*, or *EGFR-004* plasmids and treated with CHX (20 μg/mL) for the indicated times. **b** Western blotting of ABCA1 in HN6 and CAL27 cells transfected with *EGFR-001*, *EGFR-004* or control vectors, followed by treatment with 20 μmol/L MG132 for 6 h. **c** Ubiquitination level of ABCA1 protein in HN6 and CAL27 cells treated with MG132 and overexpressed with *EGFR-001*, *EGFR-004* were detected by western blotting

To further elucidate of how *EGFR-001* and *EGFR-004* regulate ABCA1 expression post-translationally, we analyzed ABCA1 protein levels in cells overexpressing these isoforms at various time points following treatment with cycloheximide. ABCA1 degradation occurred more slowly in *EGFR-001*-overexpressing cells than in *EGFR-004*-overexpressing ones, indicating a longer half-life and enhanced stability (Fig. 6a, Supplementary Fig. 10). Treating cells with the proteasome inhibitor MG132 rescued the low ABCA1 expression caused by *EGFR-004* and control transfection, whereas ABCA1 levels in *EGFR-001*-overexpressing cells remained stable (Fig. 6b), suggesting that EGFR-001 inhibits the proteasomal degradation of ABCA1. Ubiquitination of proteins plays an important role in proteasomal degradation. Furthermore, anti-ubiquitination western blotting after immunoprecipitation of ABCA1 showed that the ubiquitination level of ABCA1 was significantly reduced in *EGFR-001*-overexpressing cells (Fig. 6c). These results demonstrate that *EGFR-001* inhibits ABCA1 ubiquitination and proteasomal degradation by reducing EGFR phosphorylation, thereby increasing ABCA1 expression in OSCC cells.

### ABCA1 affects membrane fluidity and vimentin-mediated EMT in OSCC cells via cholesterol efflux pathway

**Fig. 7:**
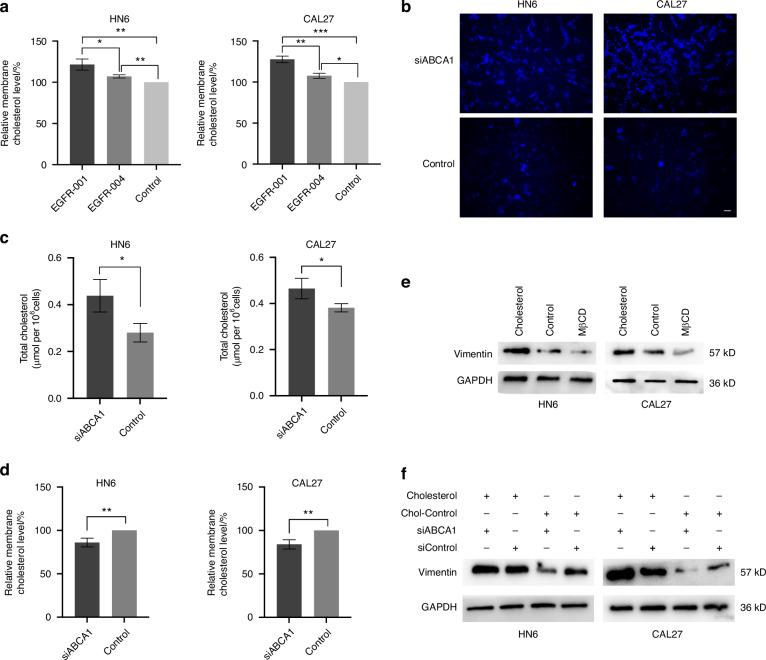
ABCA1 modulates membrane fluidity and Vimentin-mediated EMT in OSCC cells via cholesterol efflux. **a** Relative membrane cholesterol levels after overexpression of different *EGFR* isoforms. **b** Filipin staining was performed after ABCA1 was knocked down. **c** Total cholesterol contents after ABCA1 knockdown. **d** Membrane cholesterol levels were measured after ABCA1 knockdown. **e** Protein expression of Vimentin in HN6 and CAL27 cells treated with cholesterol and MβCD. **f** Effects of siABCA1 and cholesterol on the expression of Vimentin protein in HN6 and CAL27 cells. Statistical significance was assessed with Student’s *t*-test. *P < 0.05, **P < 0.01, ***P < 0.00 1

Total cholesterol levels were higher in OSCC tissues than in adjacent normal tissues (Supplementary Fig. 11a). The membrane cholesterol levels in *EGFR-001*-overexpressing OSCC cells were higher than those in EGFR-004-overexpressing ones (Fig. 7a). This suggested the positive correlation of membrane cholesterol levels with the malignancy of OSCC. Next, we assessed total cholesterol levels following ABCA1 knockdown and found that this knockdown led to significant cholesterol accumulation, as confirmed by Filipin staining (Fig. 7b, c). This contradicted the findings that both high ABCA1 expression and high cholesterol are tumor-promoting factors. However, when we measured the membrane cholesterol levels, we observed a significant decrease in cholesterol levels after ABCA1 knockdown (Fig. 7d). It has been reported that lower membrane levels of cholesterol can decrease membrane fluidity.^18^ Indeed, fluorescence-based pyrene probe assays confirmed that ABCA1 depletion reduced membrane fluidity (Supplementary Fig. 11b). These results suggest that ABCA1 influences the mobility of the plasma membrane in OSCC cells by regulating the plasma membrane levels of cholesterol.

Immunoprecipitation coupled with mass spectrometry identified vimentin as a potential ABCA1 interactor (Supplementary Fig. 11c, Supplementary Table S2). Vimentin is an important EMT marker; thus, we confirmed EMT protein expression after ABCA1 knockdown and found that ABCA1 deletion significantly reduced EMT (Supplementary Fig. 11d). Depleting plasma membrane cholesterol using methyl-β-cyclodextrin (MβCD) decreased vimentin expression, and cholesterol supplementation reversed the ABCA1 depletion-induced reduction in vimentin expression (Fig. 7e). Based on these results, we hypothesized that ABCA1 regulates vimentin through a cholesterol-dependent mechanism. To test this hypothesis, we supplemented cholesterol while simultaneously knocking down ABCA1 (Fig. 7f). Results revealed that cholesterol accumulation effectively reversed the reduction in vimentin expression induced by ABCA1 depletion.

### ASO reverses cholesterol-induced resistance in OSCC-resistant cells

**Fig. 8:**
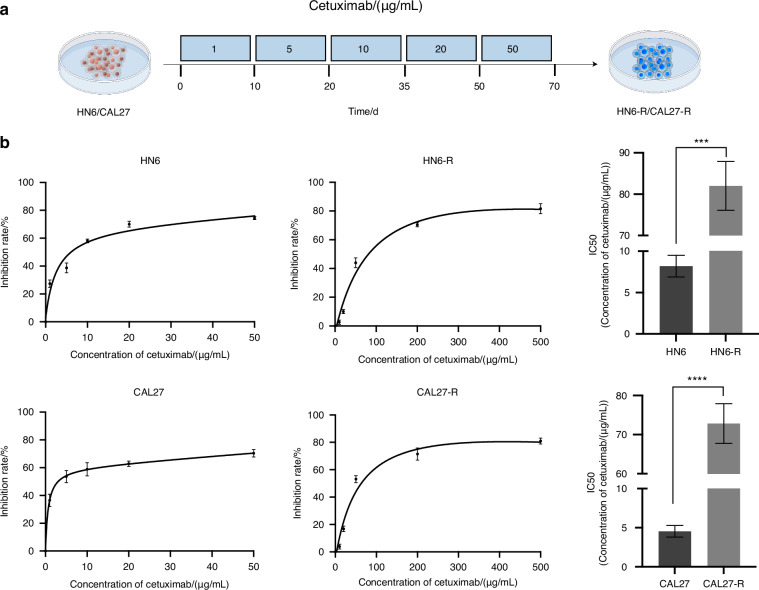
Construction of cetuximab-resistant OSCC cell lines. **a** HN6 and CAL27 cells were exposed to cetuximab to construct cetuximab-resistant HN6-R and CAL27-R cells. **b** The cell viability of HN6-R and CAL27-R cells was measured by CCK-8. Statistical significance was assessed with Student’s *t*-test. ***P < 0.001, ****P < 0.0001

To create cetuximab-resistant HN6-R and CAL27-R cell lines, parental HN6 and CAL27 cells were exposed to increasing concentrations of cetuximab, starting at 1 μg/mL, until they were able to proliferate freely in 50 μg/mL cetuximab (Fig. 8a). CCK-8 assays confirmed that these cells had successfully developed resistance to cetuximab (Fig. 8b).

**Fig. 9:**
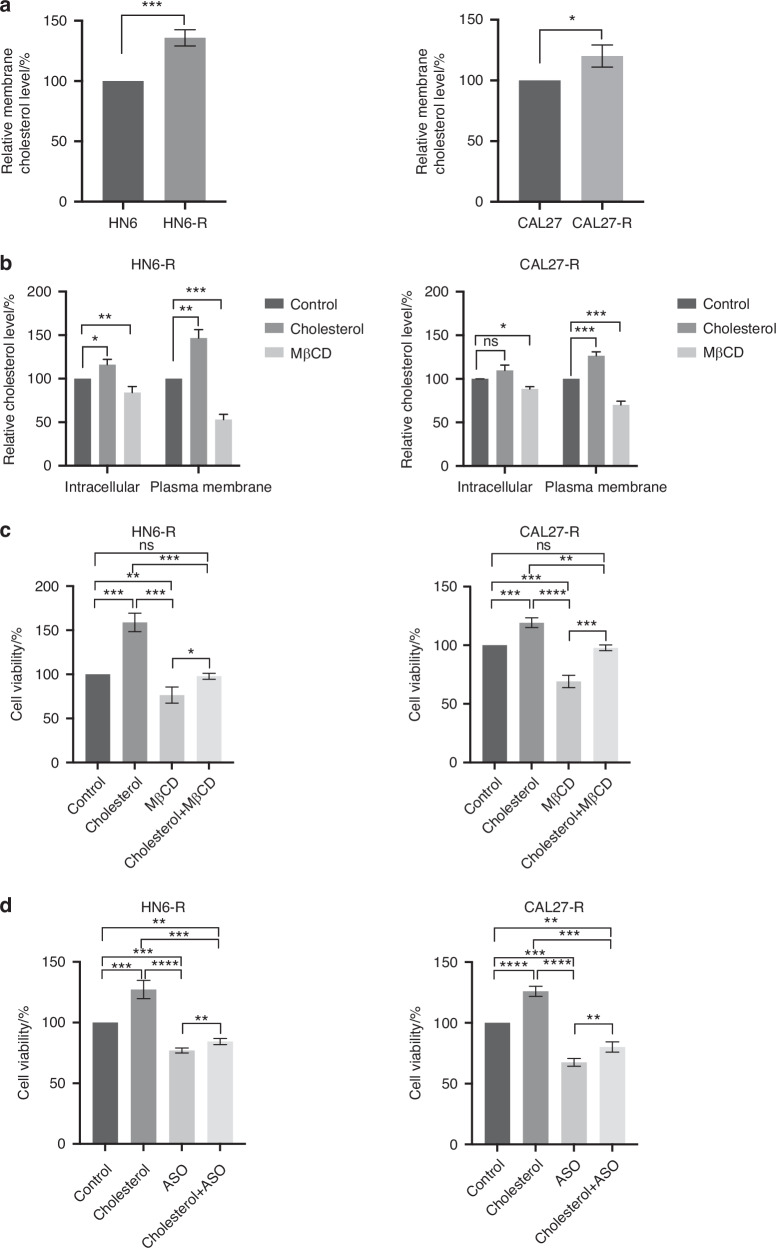
ASO counteracts cholesterol-induced cetuximab resistance. **a** Relative membrane cholesterol level in HN6, CAL27, HN6-R and CAL27-R cells. **b** Relative intracellular and plasma membrane levels of cholesterol in HN6-R and CAL27-R cells treated with 2.5 mmol/L MβCD or 10 µmol/L cholesterol. **c** After 50 µg/mL cetuximab was added, cells were treated with 2.5 mmol/L MβCD, 10 µmol/L cholesterol or 2.5 mmol/L MβCD + 10 µmol/L cholesterol for 12 h, cell viability was measured by CCK-8. **d** After 50 µg/mL cetuximab was added, cells were treated with 20 µLpermol ASO, 10 µmol/L cholesterol or 20 µLpermol ASO + 10 µmol/L cholesterol for 12 h, cell viability was measured by CCK-8. Statistical significance was assessed with Student’s t-test. “ns” *P* > 0.05, *P < 0.05, **P < 0.01, ***P < 0.001, ****P < 0.000 1

Resistant HN6-R and CAL27-R cells contained more membrane cholesterol than their parental cells (Fig. 9a). Next, we investigated the sites at which cholesterol accumulated in resistant cells. After adding cholesterol and MβCD, we observed that the change in membrane cholesterol levels was greater than that in intracellular cholesterol levels (Fig. 9b). Treating resistant cells with 50 µg/mL cetuximab and 10 µM cholesterol increased resistance, whereas MβCD reduced resistance (Fig. 9c). Subsequently, we explored the effect of the interaction between ASO and cholesterol in HN6-R and CAL27-R cells overexpressing rs737540-T. When ASO was applied to cholesterol-accumulating resistant cells, it significantly reduced their viability (Fig. 9d), demonstrating that ASO reverses cholesterol-induced drug resistance.

## Discussion

For over a decade, extensive studies have been performed on the influence of genetic variations on mRNA abundance. This work has revealed that nearly all genes harbor variations linked to expression.^19,20^ Only a fraction of GWAS signals can be attributed to eQTLs, due to various factors, such as spatiotemporal eQTL effects specific to tissues or cell types at distinct developmental stages as well as mechanisms that transcend the control of genetic variations on mRNA abundance.^21,22^ Alternative splicing regulation by genetic variants represents another crucial mechanism, albeit one that has been scarcely studied. Importantly, sQTLs are partially independent of eQTLs and contribute significantly to genetic risks for various diseases.^23,24^

**Fig. 10:**
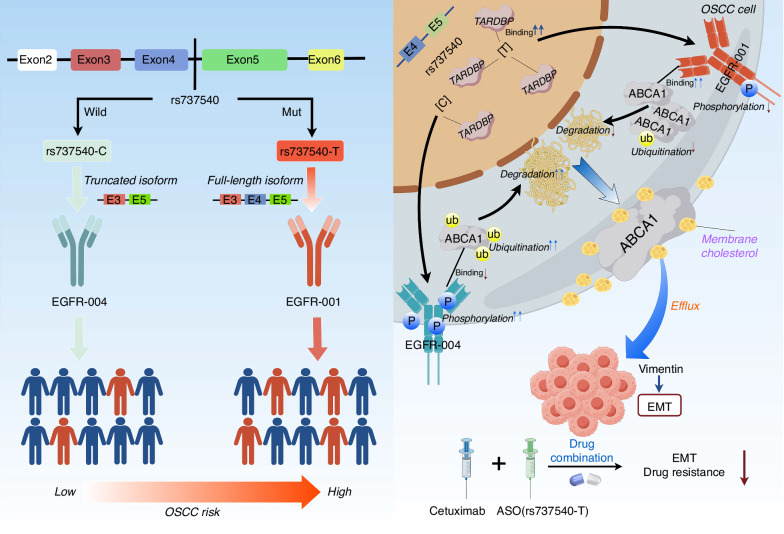
Schematic illustration of the function and mechanism of the rs737540 in OSCC risk (By Figdraw). Compared with the rs737540-C allele, the risk rs737540-T allele promotes the inclusion of exon 4 of *EGFR* mediated by TARDBP to increase the expression of the full-length *EGFR* isoform (*EGFR-001*) and decrease the expression level of the truncated *EGFR* isoform (*EGFR-004*). Compared with the *EGFR-004* subtype without exon 4, *EGFR-001* was more able to inhibit ABCA1 ubiquitination and degradation by inhibiting EGFR phosphorylation, thus promoting OSCC cell proliferation, which provided functional evidence supporting our findings in a large population that rs737540-T contributes to increased OSCC risk. Meanwhile, ABCA1 can affect membrane fluidity by regulating membrane cholesterol levels. In addition, ASO targeting rs737540-T showed a significant tumor suppressor effect. The combination of ASO and Cetuximab shows broad prospects

A critical consideration in mapping sQTLs relevant to cancer is the avoidance of confounding by tumor-specific somatic alterations. To directly address this and identify germline variants influencing splicing under conditions approximating normal physiology, we systematically identified sQTLs in normal oral epithelial adjacent to OSCC. This strategy is consistent with a previous cancer sQTL study, which demonstrated that 59.2% of its sSNPs can be calculated from GTEx lung tissues.^8^ The remaining 40.8% of sSNPs were not captured in GTEx lung tissues. The author considered that this discrepancy may arise from differences in alternative splicing patterns and allele frequencies between populations. Given these findings, we propose that the normal oral epithelial adjacent to OSCC and healthy oral epithelial tissues share the vast majority of sQTLs. We further assessed the association of sQTLs with OSCC risk. Prioritizing the most significant risk locus (rs737540), we described how the T allele reduces *EGFR* exon 4 skipping by enhancing TARDBP binding, increasing the *EGFR-001* isoform. Moreover, *EGFR-001*, compared with *EGFR-004*, reduced EGFR phosphorylation, leading to decreased ABCA1 ubiquitination and proteasomal degradation. Increased ABCA1 promoted the accumulation of cholesterol in the plasma membrane, affecting membrane fluidity and vimentin-mediated EMT (Fig. 10). Furthermore, we exploited this mechanism therapeutically using an ASO targeting the rs737540 RNA sequence to inhibit the downstream cholesterol efflux pathway and overcome resistance.

It has been reported that cholesterol efflux and esterification affect the formation of tumor cells.^25^ For example, cholesteryl ester accumulation induced by PTEN loss and PI3K/AKT activation underlies human prostate cancer aggressiveness.^26^ Even plasma cholesterol may serve as a biomarker to distinguish cancerous from precancerous stages. A study has indicated that patients with gastric cancer have significantly elevated plasma cholesterol levels compared to those with chronic superficial gastritis.^27^ And like fatty acids, cholesterol not only modulates cellular functions by altering membrane fluidity and rigidity but also affects cellular resistance to ferroptosis.^28^ In addition, long-term exposure to EGFR-TKIs in non-small cell lung cancer was shown to induce resistance characterized by the accumulation of cholesterol in the plasma membrane.^29^ Another research analyzing thirteen EGFR-expressing breast cancer cell lines for their response to EGFR-TKIs found that pharmacological depletion of cholesterol from lipid rafts decreases resistance to EGFR-TKI-induced growth inhibition. In our study, we observed that cetuximab-resistant OSCC cells accumulated cholesterol in the plasma membrane and were re-sensitized to EGFR-TKIs by membrane cholesterol inhibitors. Therefore, the targeting of cholesterol metabolism could potentially be a viable approach in cancer therapy, including targeting cholesterol synthesis, transport, or metabolites.^30,31^

By comparing *EGFR* isoforms, we identified a pathway by which resistance to EGFR-TKIs develops in OSCC, namely, ABCA1-mediated cholesterol efflux, elevating plasma membrane cholesterol. To address the challenges associated with EGFR-TKI resistance, we developed an ASO that reduces membrane cholesterol, inhibits invasion and resistance, and acts synergistically with cetuximab to achieve superior tumor inhibition. This demonstrates the feasibility of targeting sQTLs in order to treat OSCC, offering significant value for precision medicine in high-risk populations. However, similar to all drugs, oligonucleotide therapeutics carry the risk of causing unintended toxicities, which can be subdivided into hybridization-independent and hybridization-dependent effects, with the former group comprising the majority for ASOs. These toxicities stem either from the oligonucleotide chemistry itself, which impacts innate immunity, or from components of the delivery system, resulting in toxicities such as nephrotoxicity and hepatotoxicity.^32^ This is more evident in the so-called “first-generation” ASOs (i.e., containing fully phosphorothioated backbones). Subsequent “second-generation” modifications, such as the incorporation of 2′-O-methoxyethyl groups, enhanced potency and in vivo stability relative to those of first-generation ASOs, resulting in partial mitigation of certain toxicities associated with the phosphorothioate ASO class.^33^ Hybridization-dependent toxicities represent another major class of adverse effects, which include off-target effects arising from hybridization-mediated interactions with partially or fully complementary unintended RNA transcripts. Considering that the off-target effects during splicing cannot currently be accurately predicted, some research efforts have shifted toward seeking strategies to reduce the off-target activity.^34^ When alpplying ASOs, the use of appropriate in silico methods for design, using shorter ASOs, mixed-chemistry ASOs, ASO combinations, or targeted mismatches can minimize off-target effects. Regarding the delivery methods, ASOs are more specific toward on-target splice-switching when delivered via free uptake and in vivo, in contrast to in vitro delivery.^34,35^

There are certain limitations of this study, which are worth noting. First, the relatively small sample size reduced the statistical power of the sQLT and GWAS analysis, and the low frequency of the rs737540-T allele in European and American populations makes it difficult to validate the findings for it in large independent public databases such as GTEx. It is thus essential to establish a genotype-alternative splicing database for normal oral epithelium tissues with a larger sample size in order to find additional sQTLs and to conduct internal validation. Second, genetic variation exerts cell-type-specific regulation on gene transcription. However, our sQTL analysis used bulk oral epithelial tissue RNA-seq, preventing cell type-specific sQTL identification. Future studies should use cell-type-resolved RNA-seq data for sQTL mapping. Third, the development of ASOs is not the primary focus of the study described here. Accordingly, the investigation was restricted to the use of computational simulation and NCBI BLAST analysis to assess the transcriptome-wide specificity of candidate ASOs, in accordance with consensus recommendations.^36^ Thus, future research is needed to experimentally address the potential for ASOs to induce confounding effects.

## Materials and Methods

### Patients and specimens

The study has been approved by the Ethics Committee of Nanjing Medical University (Approval No. [2024]144). Written informed consent was obtained from all participating individuals. A total of 1 044 patients diagnosed with OSCC were recruited from four hospitals in Jiangsu Province, including Jiangsu Provincial People’s Hospital, Nanjing Drum Tower Hospital, Jiangsu Provincial Stomatology Hospital, and Jiangsu Provincial Cancer Hospital. Concurrently, 3 199 cancer-free patients who participated in the region’s chronic disease screening program for routine physical examination during the same period were also recruited as controls. Blood samples were collected from the morning fasting venous blood of OSCC patients and controls. Tissue samples, specifically from individuals with newly diagnosed malignant tumors, were obtained during surgery. Both the tumor and adjacent normal tissues were subjected to rigorous pathological examination by two independent pathologists to ensure that each tumor sample contained >70% tumor cells and that the adjacent normal tissue was devoid of tumor cells.

### Genotype data and RNA-seq data processing

DNA Genotyping of peripheral blood samples was conducted using the Illumina Global Screening Array (GSA). Genotyped and imputed SNP data were filtered with standard quality control criteria using PLINK (v1.9): genotyping rate >0.95, Hardy‒Weinberg equilibrium *P*-value > 1 × 10^−6^, and minor allele frequency (MAF) > 0.05. Our analysis focused specifically on autosomal chromosomal variants.

RNA-seq and its quality control procedures have been described in a prior publication.^37^ In summary, total RNA was extracted from samples following rigorous quality and quantity assessments. Transcriptome sequencing was carried out on the Illumina HiSeq 1500 platform. The resulting RNA-seq FASTQ data were cleansed using FASTQC and then aligned to the GENCODE (v.19) genome assembly with STAR (v.2.5.3a). Quantification was performed using RSEM (v.1.3.0), expressing transcripts as transcripts per million (TPM). Following stringent quality control, RNA-seq data from 81 individuals were selected for analysis of differentially expressed genes. For further sQTL analysis, DNA genotyping and RNA-seq data from 67 individuals of Chinese ancestry were included. The baseline characteristics of the 67 participants are summarized in Supplementary Table S3.

### sQTL mapping and association analysis between sQTLs and OSCC

The identification of sQTLs was achieved through the utilization of THISTLE, a transcript-level mapping method. Standard quality control measures were implemented on TPM (transcripts per million) values across all transcripts, adhering to the requirements of THISTLE.^38^ Subsequently, we adjusted the isoform-level transcriptional abundance for factors identified by VariancePartition and PEER (probabilistic estimation of expression residuals) factors.^39,40^ As outlined in the GTEx study, the number of PEER factors used for adjustment was determined based on the sample size of the dataset, with 15 factors applied for datasets with *n* < 150 samples.^6^

Our analysis focused on genes with multiple isoforms, specifically those with at least one isoform exhibiting differential expression in OSCC tissue compared with adjacent normal tissue (|log_2_FC|> 1, FDR < 0.05). The search for cis-sQTLs was restricted to SNPs located within 1 Kb of each gene on either side. Linear regression analysis was employed to detect isoform-eQTLs, adjusting for age, sex, and the first five principal components (PCs) of genetic ancestry.

The permute 1000 command in THISTLE was used to calculate the empirical *P*-value of each gene. To account for multiple testing, we applied the Benjamini‒Hochberg method to calculate gene-level false discovery rates (FDRs) using the empirical *P*-values of each gene. An FDR threshold of <0.1 was set to identify significant sQTL genes (sGenes). For variant–gene pair identification, we utilized the empirical *P*-value closest to the 0.1 FDR threshold to establish a nominal *P*-value threshold for each gene based on beta distribution parameters derived from permutations. Specifically, the nominal *P*-value threshold was calculated as F^−1^(p_t_), where F^−1^ represents the inverse cumulative distribution function.^41^ Variant-intron pairs with *P*-values below this threshold were considered significant for each gene.

To gain insights into the functional significance of sQTLs, we annotated the cis-sQTL SNPs utilizing annotated data from diverse biological databases, including SnpEff,^42^ the Roadmap Epigenomics Mapping Consortium (REMC),^43^ and POSTAR3.^44^

To investigate the potential link between sQTL SNPs and OSCC risk, we conducted a case‒control study. Using PLINK (1.9), we performed a logistic regression analysis, adjusting for variables such as age, sex, and the first five principal components (PCs). Subsequently, we selected the most significant risk locus within linkage disequilibrium (LD) blocks (r^2^ ≥ 0.8) for further validation in both population-based and experimental settings.

### Cell lines

Human OSCC cell lines (CAL27, HN6 and HN4) and human oral mucosal keratinocytes (HOKs) were obtained from the cell library of the Shanghai Institutes for Biological Sciences, Chinese Academy of Sciences. The cell lines were cultured in DMEM supplemented with 10% fetal bovine serum (FBS) and 1% antibiotics (penicillin/streptomycin) at 37 °C with 5% CO_2_. All cell lines tested negative for mycoplasma contamination.

### Reverse transcription-quantitative polymerase chain reaction (RT‒qPCR)

Total RNA was extracted from OSCC cell lines with TRIzol reagent (Sigma, USA). The RNA was reverse transcribed with a PrimeScript RT reagent kit (TaKaRa, Japan). A SYBR Premix Ex Taq Kit (Takara, Japan) was used to conduct real-time PCR experiments on an ABI QuantStudio7 quantitative PCR instrument (ABI, USA). The means of relative quantification (2^−ΔΔCt^ method) were used to calculate the fold change. Primer sequences are listed in Supplementary Table S4.

### Minigene splicing assay

CAL27 and HN6 cells were transfected with allele-specific *EGFR* minigene plasmids. Total RNA was isolated with TRIzol reagent and reverse transcribed. To detect splicing alterations, minigene-specific cDNA was amplified by PCR and separated on 2% agarose gel electrophoresis. The amplified minigene-specific cDNA was quantitatively detected by RT‒qPCR.

### Construction of plasmids and siRNAs

*EGFR* minigenes containing exon 3 to exon 5 fragments with rs737540-C or -T alleles were subcloned into pcDNA3.1(-) (Genechem, Shanghai).

The cDNAs of the *EGFR-001* (*ENST00000275493*) and *EGFR-004* (*ENST00000455089*) were subcloned into the pcDNA3.1(-) vector (GeneChem, Shanghai).

Small interfering RNAs (siRNAs) targeting *TARDBP and ABCA1* and a nontargeting siRNA control were purchased from RiboBio (China). For transient transfections, jetPRIME^®^ DNA & siRNA Transfection Reagent (Polyplus, France) was used following the manufacturer’s instructions. The siRNA sequences are listed in Supplementary Table S4.

### RNA immunoprecipitation (RIP) assay

Transfected cells were washed twice with ice-cold PBS and then lysed in ice-cold wash buffer. Cell lysis was performed in cold wash buffer (1× PBS, 0.5% SDS, 0.5% NP-40 and 0.5% sodium deoxycholate) supplemented with a 400 U/mL RNase inhibitor (Takara, Japan) and protease inhibitor cocktail (Bimake, China) and incubated on ice for 30 min. RQ1 RNase-Free DNase (Promega, USA) was added to a final concentration of 0.1 U/μL, and incubated in a heat block for 30 min at 37 °C. Then, the mixture was vibrated vigorously and centrifuged (13 000 × *g* at 4 °C, 15 min) to remove cell debris.

For immunoprecipitation, the supernatant was incubated overnight at 4 °C with 6 μg TARDBP antibody (Proteintech, China) or control IgG antibody (ABclonal, China). After incubation, the beads were washed twice with RIP wash buffer. The RNA was purified using phenol:chloroform:isopentyl alcohol (25:24:1, pH<5). Coprecipitated RNAs were detected by qPCR (ABI QuantStudio 5) using probes targeting TARDBP at rs737540.

### RNA pull-down assay

RNA pull-down assay was performed using a PureBinding™ RNA‒Protein pull-down Kit (GENESEED, China). A biotinylated fragment containing 1 001 bp at the rs737540 site was commercially synthesized. Then, biotinylated RNA (50 pmol) was incubated with cellular protein extracts from CAL27 and HN6 cells and 50 μL of streptavidin magnetic beads. The beads bound to the proteins were washed three times with the wash buffer. Furthermore, 50 µL of capture buffer and loading buffer were added, and the reaction was performed at 100 °C for 10 min for protein capture. The samples were stored at −80 °C until use for western blotting.

### Western blotting

Radioimmunoprecipitation assay (RIPA) lysis buffer (Beyotime, China) was used for total protein extraction. Proteins of varying molecular weights were separated using SDS–PAGE gels ranging from 8% to 12% and transferred to PVDF membranes (Merck Millipore, Germany). Membranes were immersed in 10% skim milk for 2 h, followed by an incubated with primary antibodies overnight at 4 °C, as indicated in Supplementary Table S5. After incubation with a secondary antibody at room temperature for 1 h, signals were visualized using the High-sig ECL Western Blotting Substrate (Tanon, Shanghai, China).

### Construction of ASO

Synthesized by QianMoBio Co., Ltd (Nanjing, China), the second-generation ASO targeting rs737540 was designed as a 20-mer centered on the SNP locus (±10-nt flanking sequence). Its phosphorothioate backbone and gapmer architecture (2’-MOE-modified wings with central DNA gap) confer enhanced stability and specificity, with unique target validation confirmed by comprehensive BLAST analysis (NCBI) against the reference genome. The ASO sequence is provided in Supplementary Table S4.

### Calcein/PI cell viability assay

Transfected cells were inoculated into 96-well plates at appropriate densities. After 24 h of culture, 100 μL of 1% cetuximab was added to each well. Subsequently, after 24 to 72 h of further culture, 100 μL of calcein AM/PI detection solution was added to each well, and the plates were incubated in a dark environment at 37 °C for 30 min. The resulting staining effect was then observed under a fluorescence microscope.

### Animal Experiment

All experimental procedures were approved by the Animal Ethics and Welfare Committee of Nanjing Medical University. BALB/c nude mice (4–6 weeks old, female) were purchased and raised at the Animal Core Facility of Nanjing Medical University. The mice in each cage were randomly assigned to control or treatment groups. All animal experiments are conducted in accordance with appropriate ethical standards and national guidelines. *EGFR-001*, *EGFR-004*, or Control cells were suspended in sterile PBS and injected subcutaneously into the armpits of five mice. After four weeks, all mice were euthanized and tumor volume was measured, tumor volume (mm^3^) = width (mm^2^) × length (mm)/2. Tumors were collected for IHC staining.

### Immunohistochemistry (IHC)

The sections were fixed with 4% paraformaldehyde at room temperature for 24 h, then dehydrated in a gradient ethanol series and embedded in paraffin. After dewaxing and hydration, antigen repair was performed by heating citrate buffer at 95 °C (pH 6.0) for 20 min. Then 3% hydrogen peroxide was added for 25 min to remove endogenous peroxidase. The tissue was incubated with goat serum at room temperature for 30 min, and then mixed with anti-ABCA1 (DF8233, Affinity Biosciences), anti-Ki67 (27309-1-AP, Proteintech), anti-EGFR (AF6043, Affinity Biosciences), and anti-pEGFR (AF3048, Affinity Biosciences) antibodies were incubated at 4°C overnight. After rinsing with PBS, the slices were incubated with biotin secondary antibody at room temperature for 30 min and then incubated with avidin-biotin complex (ABC) reagent for another 30 min. The antigen-antibody complex was colored using DAB substrate and was restained with hematoxylin. Finally, the slices are dehydrated, transparent, and sealed, then viewed and photographed under an optical microscope.

### In vitro cell proliferation assays

Colony formation and CCK-8 assays were performed to detect the proliferative ability of the cells. For the colony formation assay, the transfected cells were cultured in 6-well cell culture plates at a density of 1 000 cellsperwell and incubated in complete medium at 37 °C. After 7-10 days, the cells were washed with PBS three times, fixed with 100% methanol and stained with crystal violet (Solarbio, China) at room temperature for 30 min. The number of colonies in each well was counted. Finally, the average value of three repeated data points was calculated.

For the CCK-8 assay, the transfected cells were cultured in 96-well plates at a density of approximately 3 000 cells per well for 12, 24, and 36 h. At each time point, CCK-8 solution (GLPBIO, USA) was added to each well, and the absorbance at 450 nm was measured by a microplate reader. Finally, the average value of three repeated data points was calculated.

### In vitro cell migration and invasion assays

For the migration assay, a wound healing assay was performed. The transfected cells were cultured in a six-well plate until the density reached 90%–100%. A sterile 200 µL yellow pipette tip was gently scratched vertically through the center of the well. Images were taken with the same microscope at 0 and 24 days postwounding. The wound healing rate was analyzed by ImageJ.

For the invasion assay, the transfected cells were resuspended adequately in serum-free DMEM. Transwell chambers (Corning, USA) with 100 μL of Matrigel at the bottom surface were placed on a 24-well plate for the invasion assay. The Matrigel was composed of Matrigel Basement Membrane Matrix (Corning) and DMEM at a ratio of 1:9. DMEM containing 15% fetal bovine serum was added to the lower chamber, and a transfected cell suspension (density of 1 × 10^5^) was inoculated into the upper chamber. After incubation for 24–48 h, the cells on the bottom membrane were fixed with paraformaldehyde and then stained with 0.1% crystal violet for 20 min. Images of the visual fields were randomly taken for further analysis.

### Co-immunoprecipitation (Co-IP) assay

The Co-IP procedure was performed following the manufacturer’s instructions using an IP/Co-IP Kit (Elabscience, China). Initially, 1 mL of cell lysate was used to lyse approximately 1 × 10^7^ OSCC cells. Afterward, 500 μL of IP antibody diluent was bound to Protein A/G magnetic beads at 37 °C for 30 min. Subsequently, the antigen/antibody complex was incubated with 1 mL of protein-rich cellular lysates at 4 °C for 2 h. The magnetic beads were then thoroughly washed 4 times with PBST buffer, and the immunoprecipitated complexes were separated from the beads for further analysis via western blotting.

### Ubiquitination Assay

Cultured cells were treated with 20 μmol/L MG132 (HY-13259, MCE, USA) for 6 h, followed by lysis in IP lysis buffer supplemented with protease and phosphatase inhibitors on ice for 30 min. The lysates were then incubated overnight at 4 °C with either an anti-ABCA1 antibody (DF8233, Affinity Biosciences) or IgG (30000-0-AP, Proteintech) with constant rotation. Protein A/G magnetic beads were subsequently added to the mixture and incubated at 4 °C for 2 h. The beads were then boiled in SDS loading buffer and subjected to western blotting analysis. An anti-ubiquitin antibody (#3936, Cell Signaling Technology) was used to detect the ubiquitination of ABCA1.

### Quantification of plasma membrane cholesterol

The total cholesterol in the cells was extracted using a methanol/chloroform mixture (1:2, v/v) under ultrasonic treatment. Following extraction, cholesterol levels were quantified using the Amplex Red Cholesterol Detection Kit. Intracellular cholesterol levels were assessed after treatment with cholesterol oxidase. The cholesterol level in the cell membrane was calculated by subtracting the intracellular cholesterol level from the total cellular cholesterol level.

### Cell membrane fluidity quantification

According to the manufacturer’s protocol (Membrane Fluidity Kit, Abcam), membrane fluidity was measured by flow cytometry using a fluorescent labeling method. HN6 and CAL27 cells were incubated with 2 µmol/L PDA and 0.08% Pluronic F-127 for 1 h. PDA fluorescence was assessed by exciting the samples at 350 nm and collecting emission readings at 400 nm (for monomer) and 470 nm (for excimer). The relative membrane fluidity was determined as the ratio of excimer fluorescence to monomer fluorescence.

### Filipin Staining

According to the manufacturer’s protocol (Filipin III, MedChem Express), the cells were fixed for 10 min, washed with TBST, and incubated with Filipin III (50 μg/mL in TBS) at room temperature for 1 h away from light. After washing with TBST, the cover glass was separated and installed in an aqueous sealer. Images were acquired using fluorescence microscopy.

### Statistical analysis

Data are presented as mean ± SD. Differences between two groups and among three or more groups were assessed using two-tailed Student’s *t*-tests and analysis of variance (ANOVA), respectively. For analysis involving multiple comparisons, we used the Benjamini-Hochberg method to adjust the *P*-values to control the false discovery rate. Significance levels: ns for no significant difference, * for *P* < 0.05, ** for *P* < 0.01, *** for *P* < 0.001, **** for *P* < 0.000 1). All statistical analysis were performed using R (version 4.1.0). *P* < 0.05 was considered to indicate statistical significance.

## Supplementary information

Supplemental Figures and Tables

## Data Availability

The datasets used during the current study are available from the corresponding author on reasonable request.
