# Correction: Antisense oligonucleotide targeting TARDBP-EGFR splicing axis inhibits progression of oral squamous cell carcinoma through ABCA1-regulated cholesterol efflux

**DOI:** 10.1038/s41368-026-00434-7

**Published:** 2026-03-04

**Authors:** Nan Ni, Moxu Wang, Zhiran Yuan, Leqi Zhang, Jilin Cai, Qingqing Du, Pengcheng Li, Chang Gao, Hanwen Zhang, Yuancheng Li, Hua Yuan

**Affiliations:** 1 Jiangsu Key Laboratory of Oral Diseases, Nanjing Medical University, Nanjing, China; 2 Department of Oral and Maxillofacial Surgery, Affiliated Hospital of Stomatology, Nanjing Medical University, Nanjing, China; 3 Department of Oral and Dental Implantology, Affiliated Hospital of Stomatology, Nanjing Medical University, Nanjing, China; 4 Central Research Laboratory, Jiangsu Key Laboratory of Molecular Biology for Skin Diseases and STIs, Institute of Dermatology, Chinese Academy of Medicine Sciences and Peking Union Medical College, Nanjing, China; 5 Jiangsu Province Engineering Research Center of Stomatological Translational Medicine, Nanjing, China; 6 Jiangsu Key Lab of Cancer Biomarkers, Prevention and Treatment, Collaborative Innovation Center for Cancer Personalized Medicine, Nanjing Medical University, Nanjing, China

**Keywords:** Cancer epidemiology, Oral cancer

Correction to: *International Journal of Oral Science* 10.1038/s41368-025-00402-7, published online 16 January 2026

Following publication of the original article [1], it was discovered that the Western blot images for two cell lines in Fig. 6b are unintentionally duplicated. The correct figure was provided by the authors in the final version submitted for review, and the error was caused by typesetting mistake.

Fig. 6 has been corrected from:

**Fig. 6:**
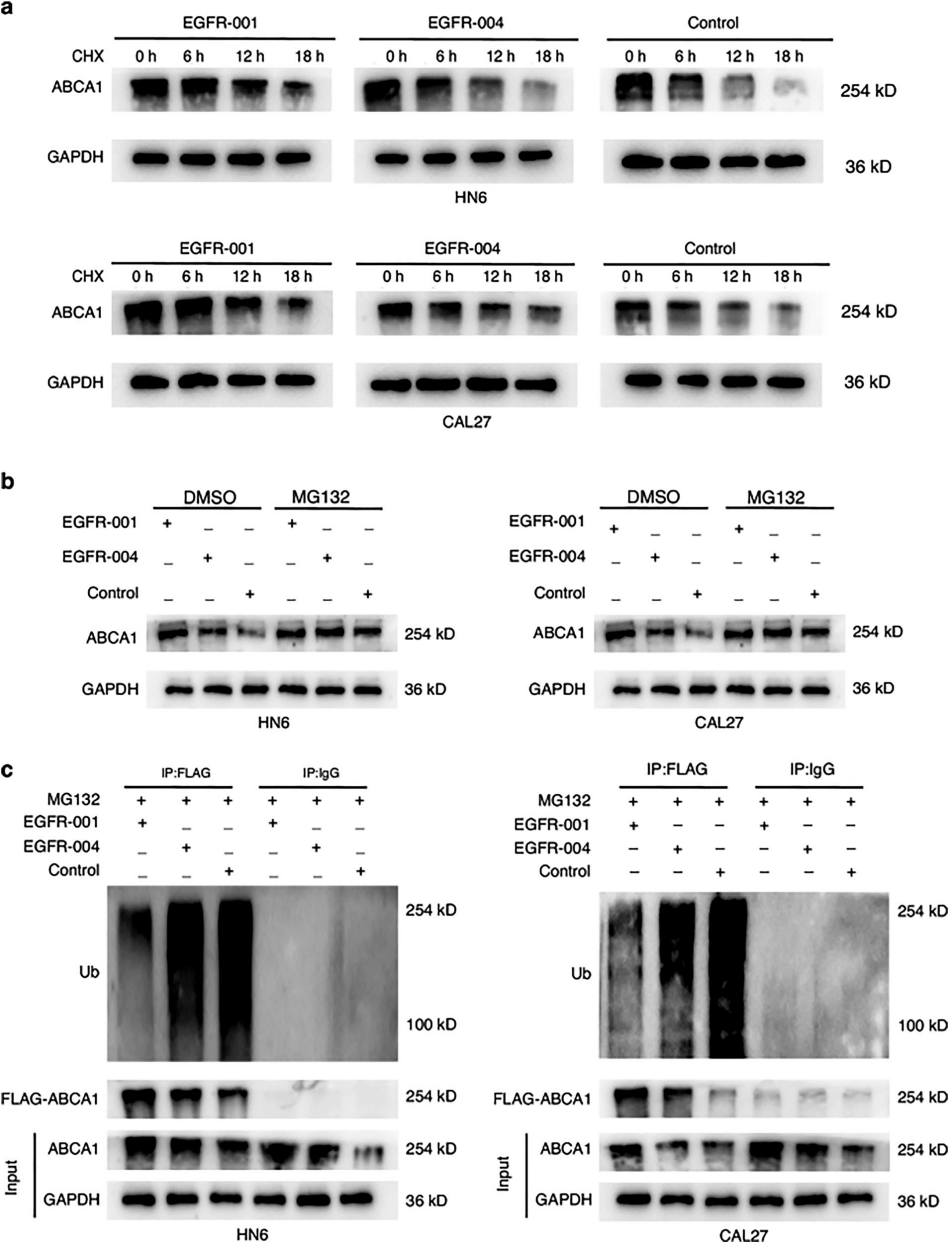
Ubiquitination levels of different EGFR isoforms. **a** Western blotting showing the ABCA1 protein in CAL27 and HN6 cells transfected with control, *EGFR-001*, or *EGFR-004* plasmids and treated with CHX (20 μg/mL) for the indicated times. **b** Western blotting of ABCA1 in HN6 and CAL27 cells transfected with *EGFR-001*, *EGFR-004* or control vectors, followed by treatment with 20 μmol/L MG132 for 6 h. **c** Ubiquitination level of ABCA1 protein in HN6 and CAL27 cells treated with MG132 and overexpressed with *EGFR-001*, *EGFR-004* were detected by western blotting

To:

**Fig. 6:**
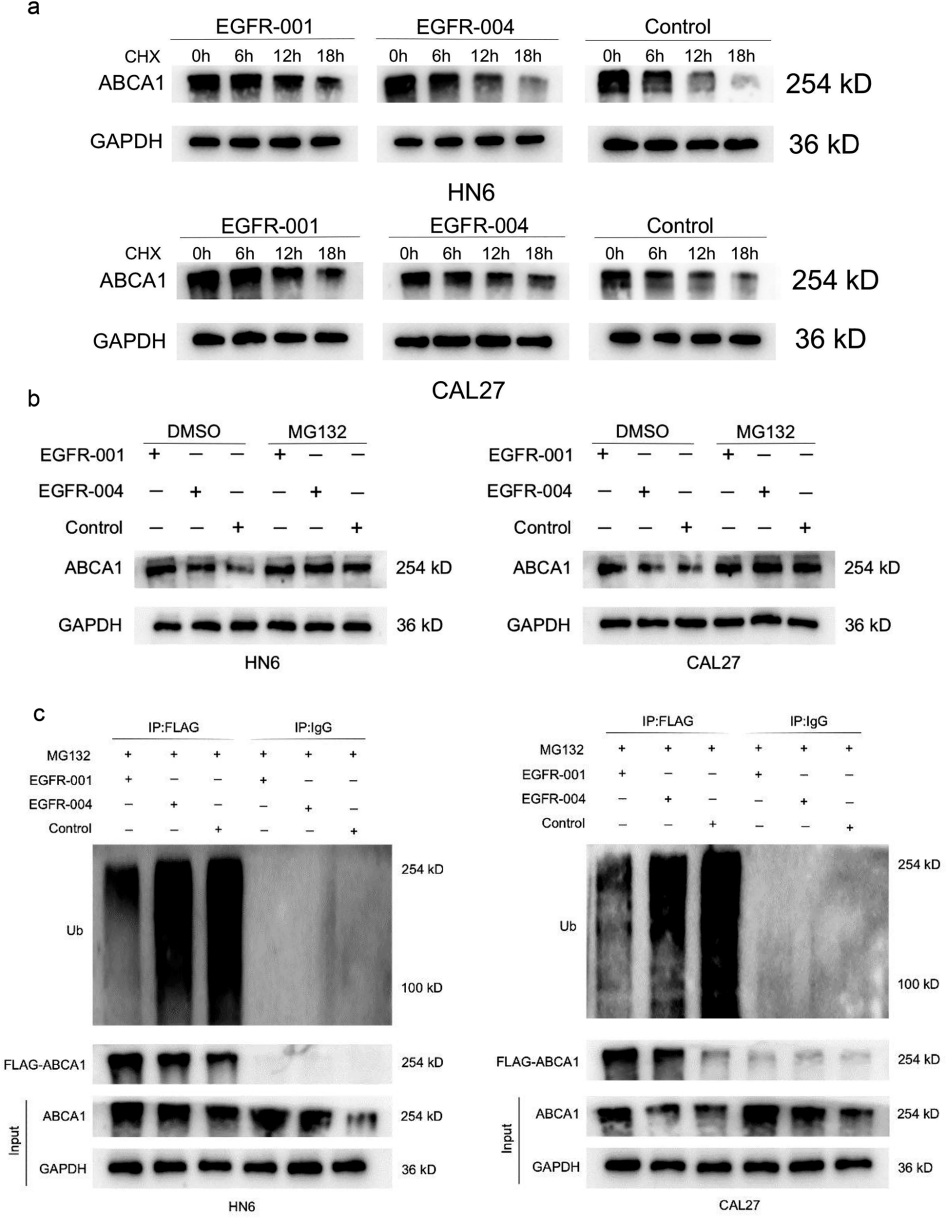
Ubiquitination levels of different EGFR isoforms. **a** Western blotting showing the ABCA1 protein in CAL27 and HN6 cells transfected with control, *EGFR-001*, or *EGFR-004* plasmids and treated with CHX (20 μg/mL) for the indicated times. **b** Western blotting of ABCA1 in HN6 and CAL27 cells transfected with *EGFR-001*, *EGFR-004* or control vectors, followed by treatment with 20 μmol/L MG132 for 6 h. **c** Ubiquitination level of ABCA1 protein in HN6 and CAL27 cells treated with MG132 and overexpressed with *EGFR-001*, *EGFR-004* were detected by western blotting

The original article [1] has been updated.
