# Supplementary material for: Antisense oligonucleotide targeting TARDBP-EGFR splicing axis inhibits progression of oral squamous cell carcinoma through ABCA1-regulated cholesterol efflux
Source: Int J Oral Sci. 2026 Jan 16;18:10. doi: 10.1038/s41368-025-00402-7 (PMC12808199; doi:10.1038/s41368-025-00402-7)
Supplement: Supplementary file 1 — Supplemental Figures and Tables [file 41368_2025_402_MOESM1_ESM.docx]

**Supplemental Figures**

**
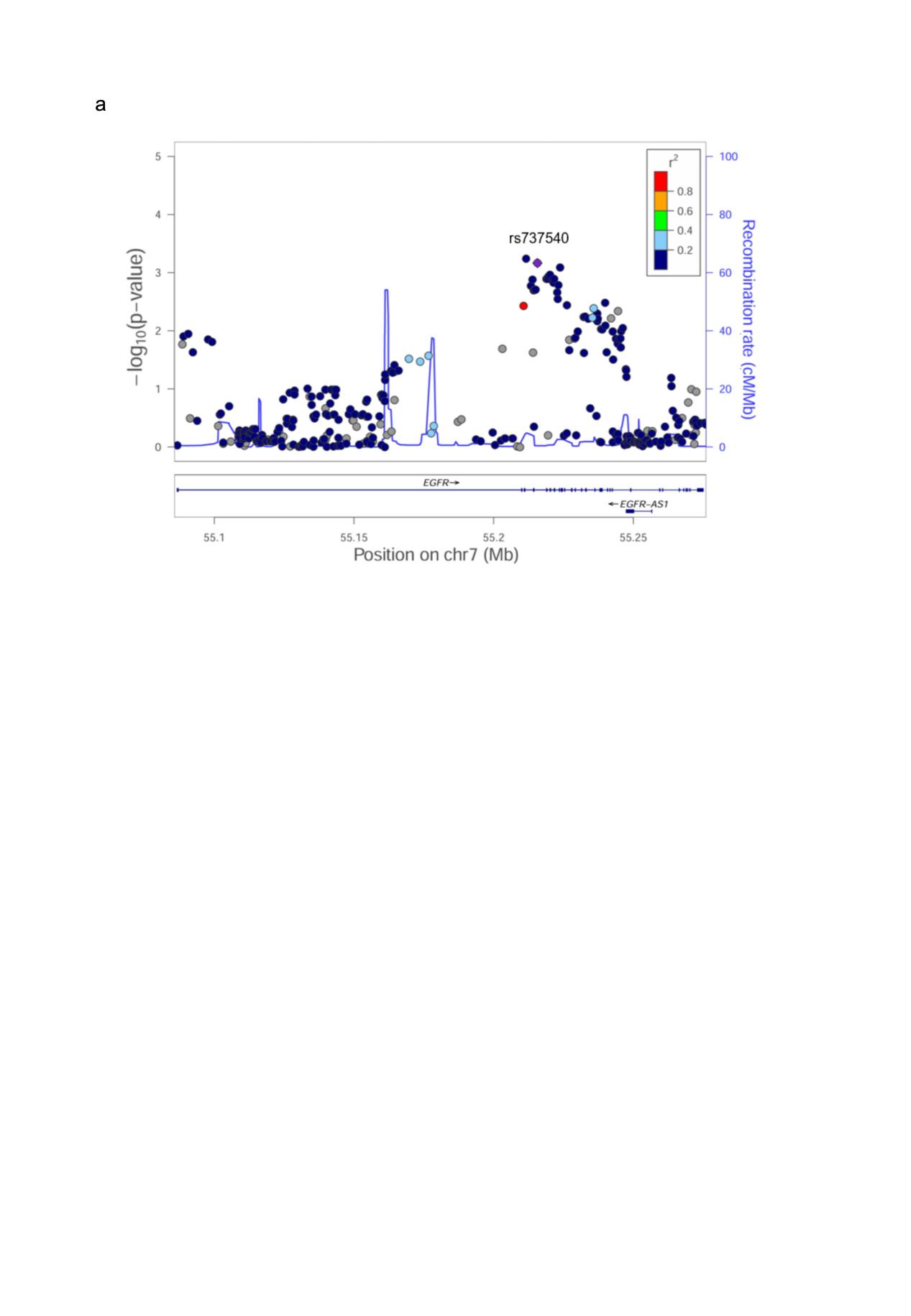
**

**Supplemental Figure 1.** Regional plot of rs737540.


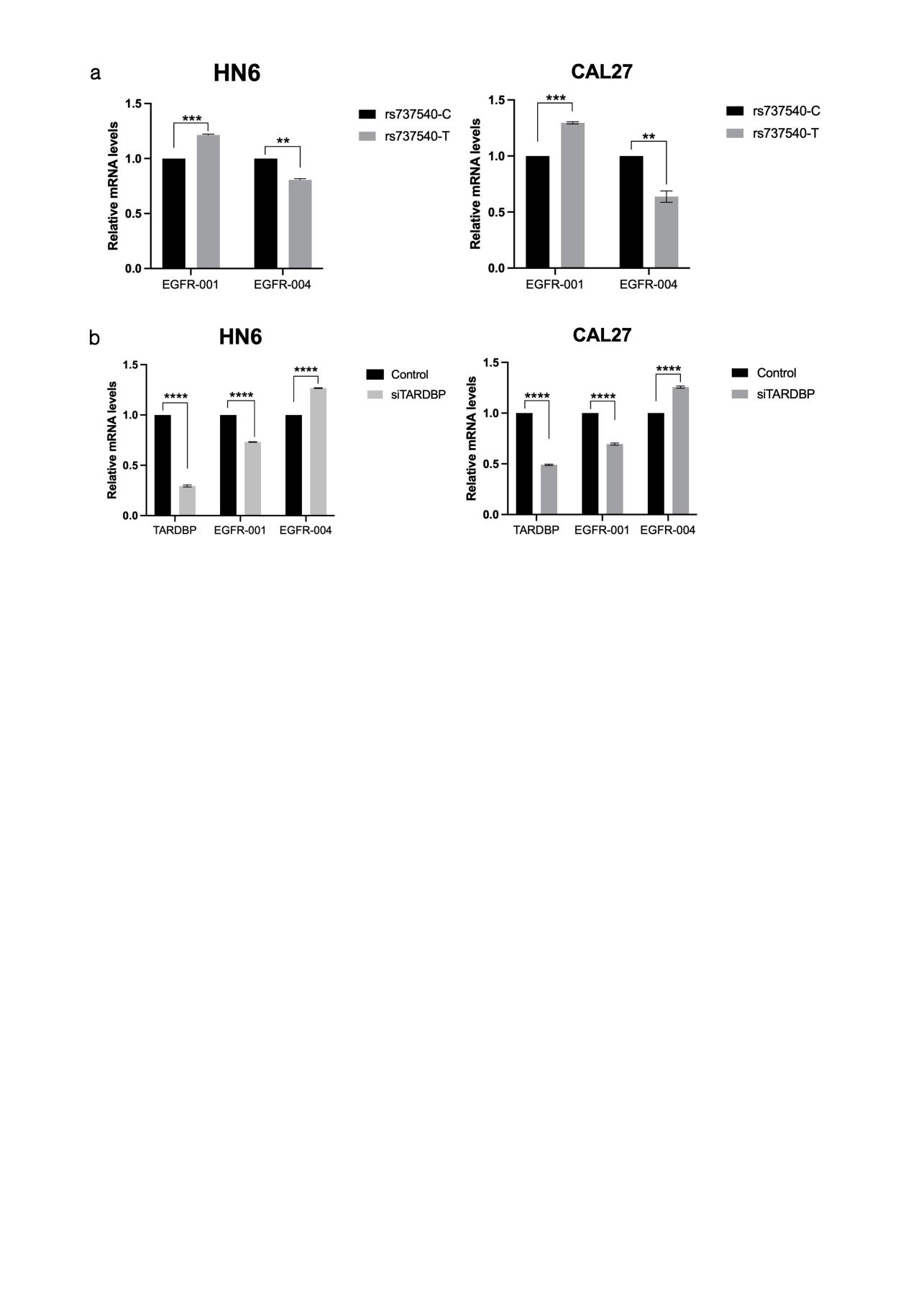


**Supplemental Figure 2.** TARDBP binds rs737540-T to promote EGFR splicing. (a) rs737540-T led to lower *EGFR-004* levels and higher *EGFR-001* levels compared with rs737540-C. (b) RT‒qPCR assays confirmed the effects of TARDBP on both EGFR-001 and EGFR-004 expression levels. Data are presented as the mean ±SD from three independent experiment. Statistical significance was assessed with Student's t-test. ** *P* < 0.01, *** *P* < 0.001, **** *P* < 0.0001.


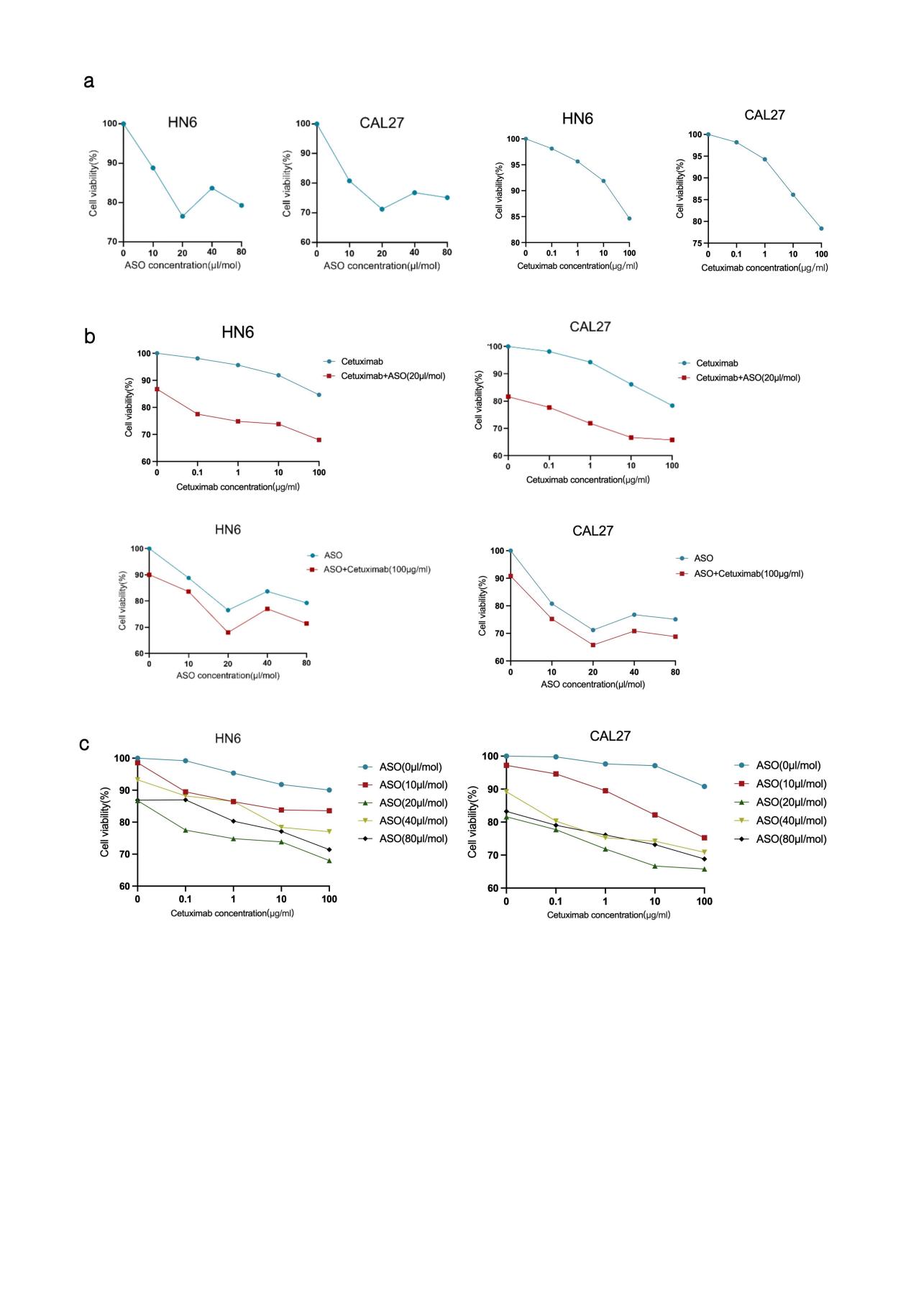


**Supplemental Figure 3.** Effect of cetuximab and/or antisense oligonucleotides. (a) Inhibitory effect of ASO and cetuximab at different concentrations on OSCC cells. (b) The effect of ASO combined with cetuximab is better than that of cetuximab or ASO alone. (c) The combined administration of cetuximab and ASO at different concentrations showed that when ASO concentration was 20μg/mL and cetuximab concentration was 100μL/mol, the inhibitory effect on OSCC cells was the most obvious.


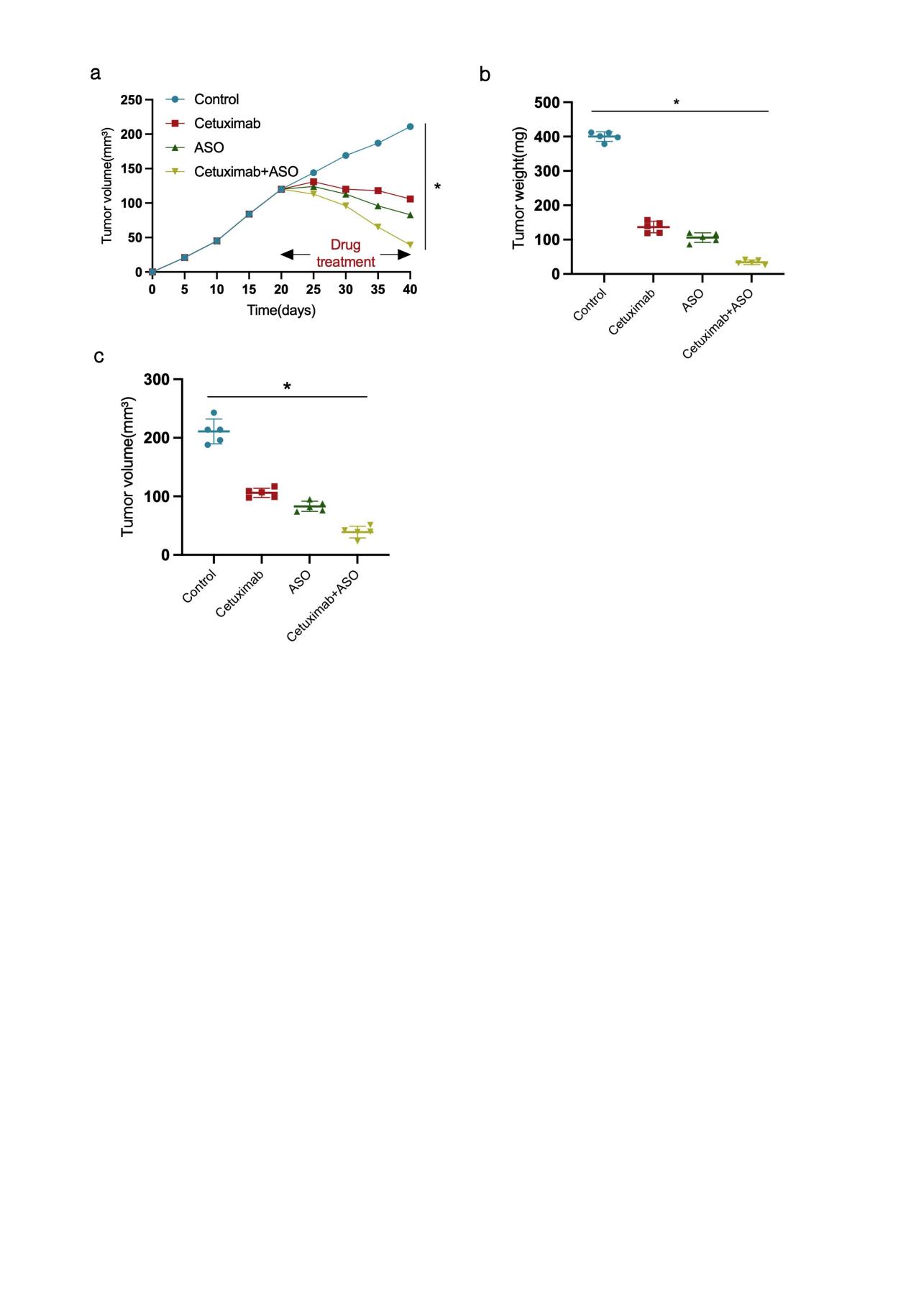
**Supplemental Figure 4.** Therapeutic effects of cetuximab and/or antisense oligonucleotides *in vivo*. Tumor volume (a) and weight (b) were measured, with pairwise comparisons performed between groups. The largest *P*-value among them was marked. (c) Quantification of tumor volume in different groups on day 40. Statistical significance was assessed with Student's t-test. * *P* < 0.05.


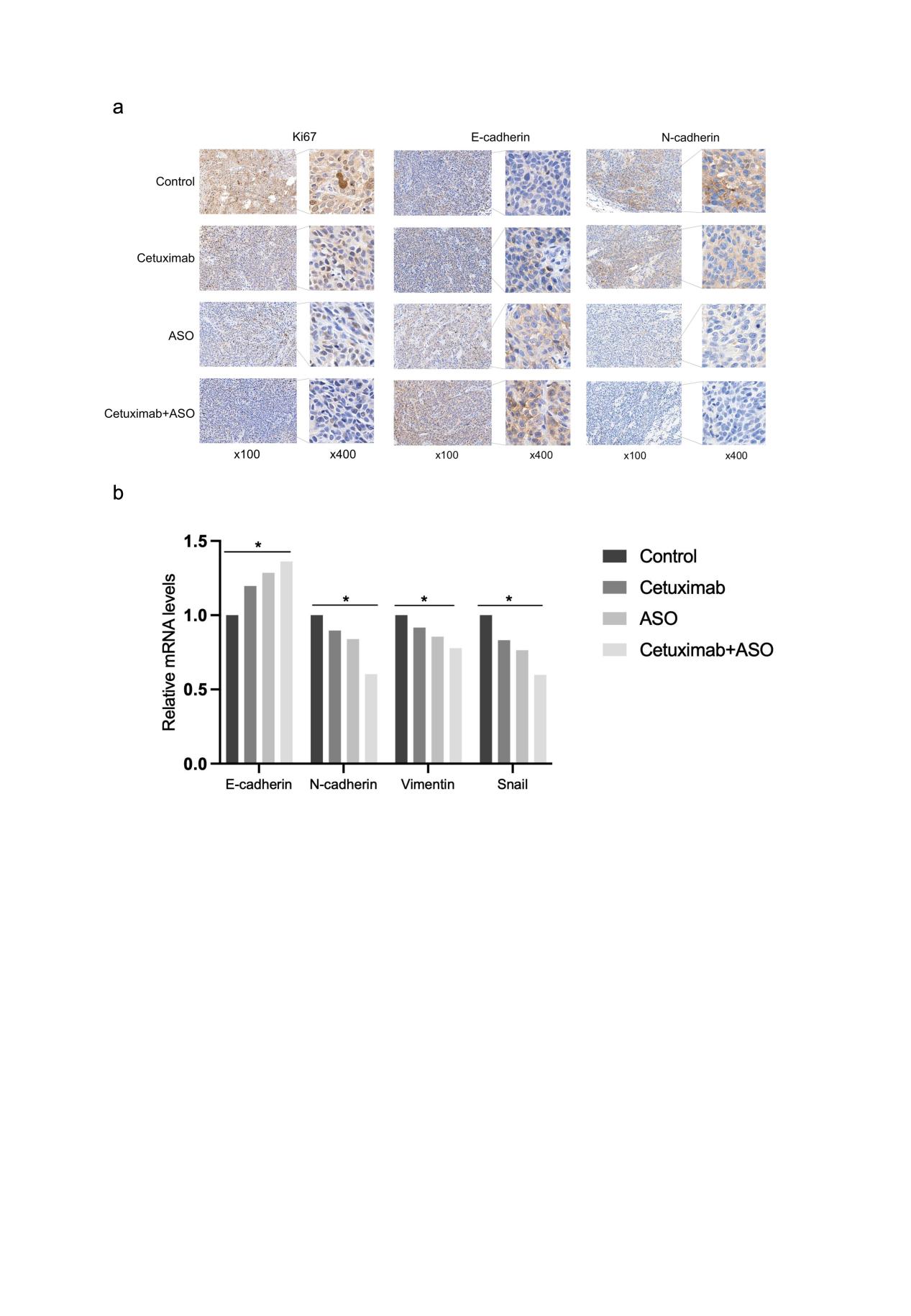


**Supplemental Figure 5.** Expression of EMT markers. (a) Immunohistochemistry (IHC) staining of Ki67, E-cadherin and N-cadherin. Scale bars, 100 μm (left), 25 μm (right). (b) RT-qPCR assays detected the expression levels of EMT markers in each group. Statistical significance was assessed with Student's t-test. * *P* < 0.05.


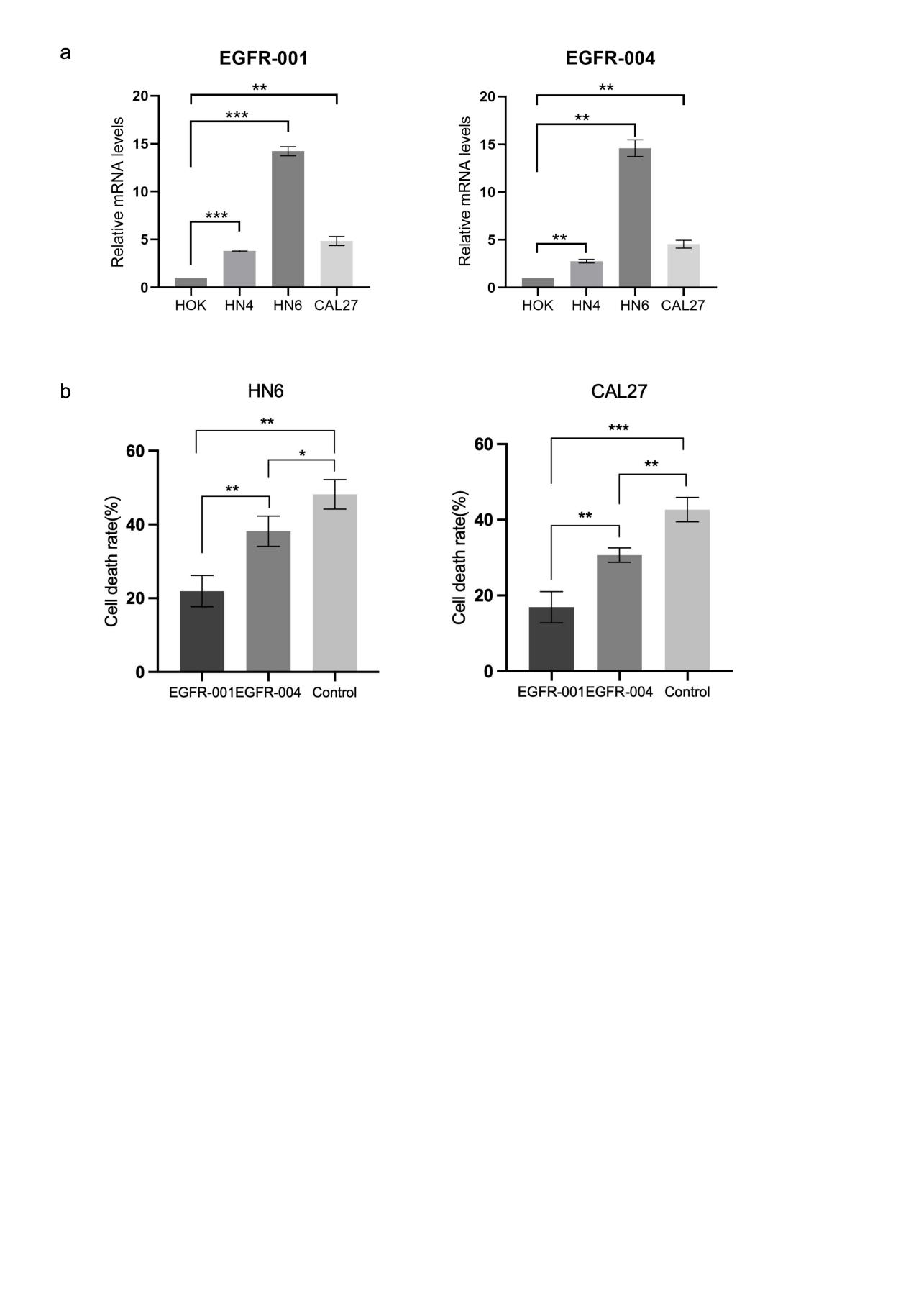


**Supplemental Figure 6.** RT-qPCR assays showed the expression levels of *EGFR-001* and *EGFR-004* in HOK, HN4, HN6 and CAL27 cells. *P*-values were adjusted using the Benjamini-Hochberg method. Statistical significance was assessed with Student's t-test. ***P* < 0.01, ****P* < 0.001.


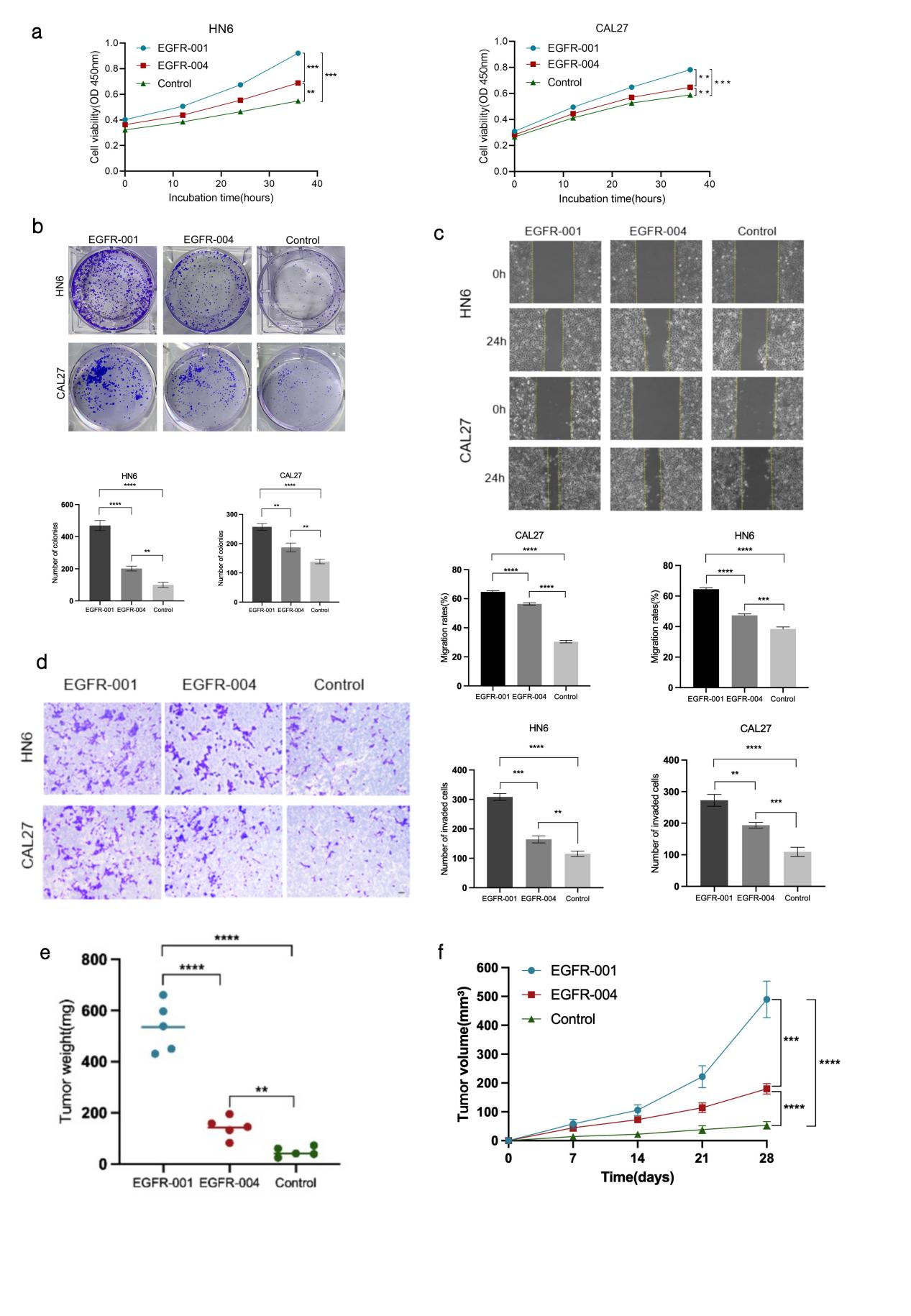


**Supplemental Figure 7.** The differential effects of EGFR isoforms on tumor cells. (a) The effects of *EGFR-001* and *EGFR-004* overexpression on the *in vitro* proliferation of HN6 and CAL27 cells was determined by CCK-8 assays. Images of (b) Colony formation assays, (c) Wound healing assays and (d) Transwell assays of HN6 and CAL27 cells transfected with vectors overexpressing EGFR-001 or EGFR-004. The data are presented as the means ± SDs from three independent experiments. Scale bars, 100 μm. The weight (e) and volume (f) of the tumors from xenograft nude mice model are measured. Statistical significance was assessed with Student's t-test. ** *P* < 0.01, *** *P* < 0.001, **** *P* < 0.0001.


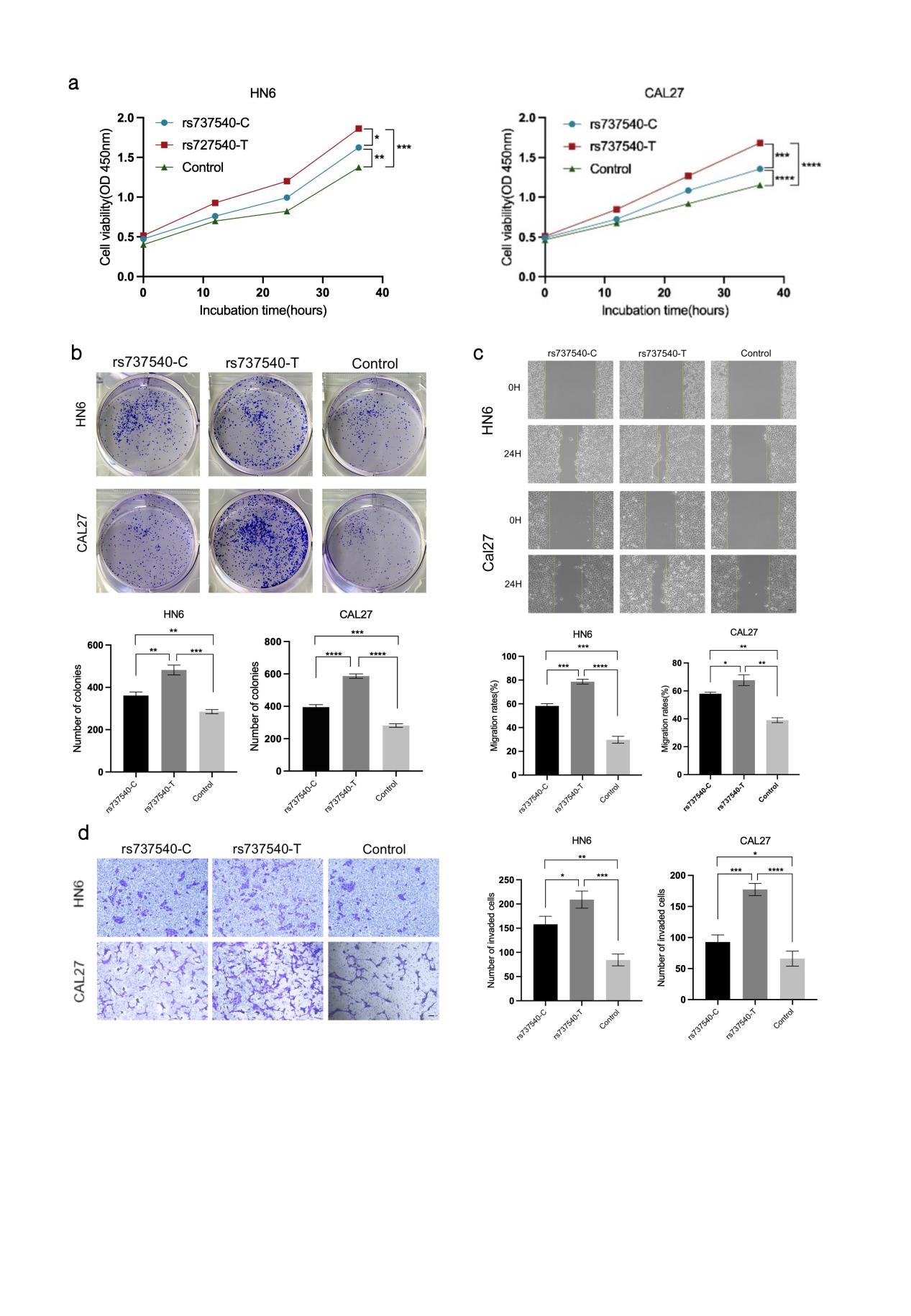
**Supplemental Figure 8.** The differential effects of rs737540 genotypes on tumor cells. (a) The effects of rs737540-C and rs737540-T overexpression on the *in vitro* proliferation of HN6 and CAL27 cells was determined by CCK-8 assays. Images of (b) Colony formation assays, (c) Wound healing assays and (d) Transwell assays of HN6 and CAL27 cells transfected with vectors overexpressing rs737540-T or rs737540-C. The data are presented as the means ± SDs from three independent experiments. Scale bars, 100 μm. Statistical significance was assessed with Student's t-test. * *P* < 0.05, ** *P* < 0.01, *** *P* < 0.001, **** *P*< 0.0001.


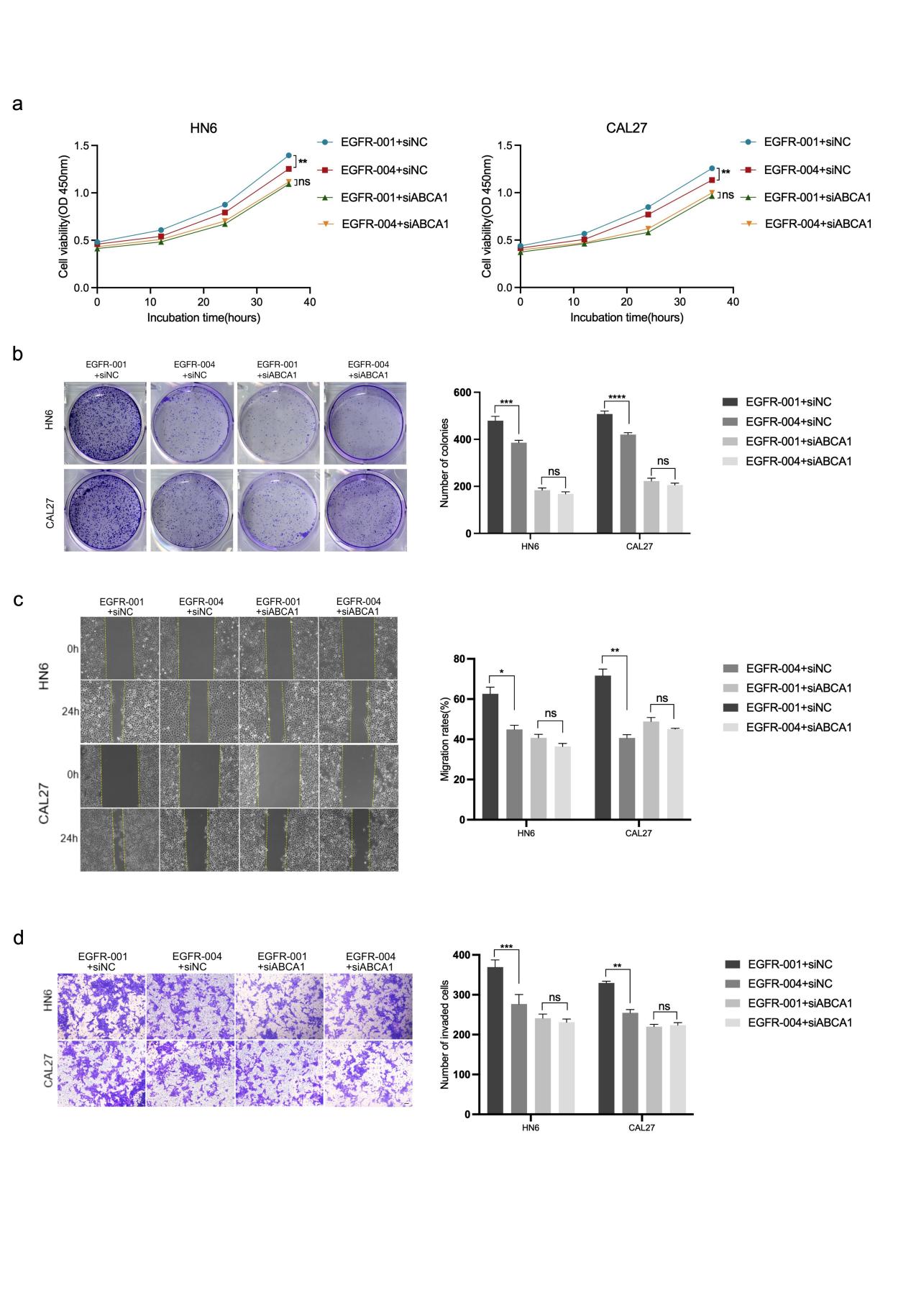


**Supplemental Figure 9.** siABCA1 significantly mitigated the differences in cell proliferation, colony formation, migration, and invasion promoted by *EGFR-001* and *EGFR-004*. Images of CCK-8 (a), Colony formation assays (b), Wound healing assays (c) and Transwell assays (d) of HN6 and CAL27 cells co-transfected with si-NC or si-ABCA1 and EGFR-001 or EGFR-004-expressing vectors in combination. The data are presented as the means ± SDs from three independent experiments. Scale bars, 100 μm. Statistical significance was assessed with Student's t-test. “ns” *P* > 0.05, * *P* < 0.05, ** *P* < 0.01, *** *P* < 0.001, **** *P* < 0.0001.


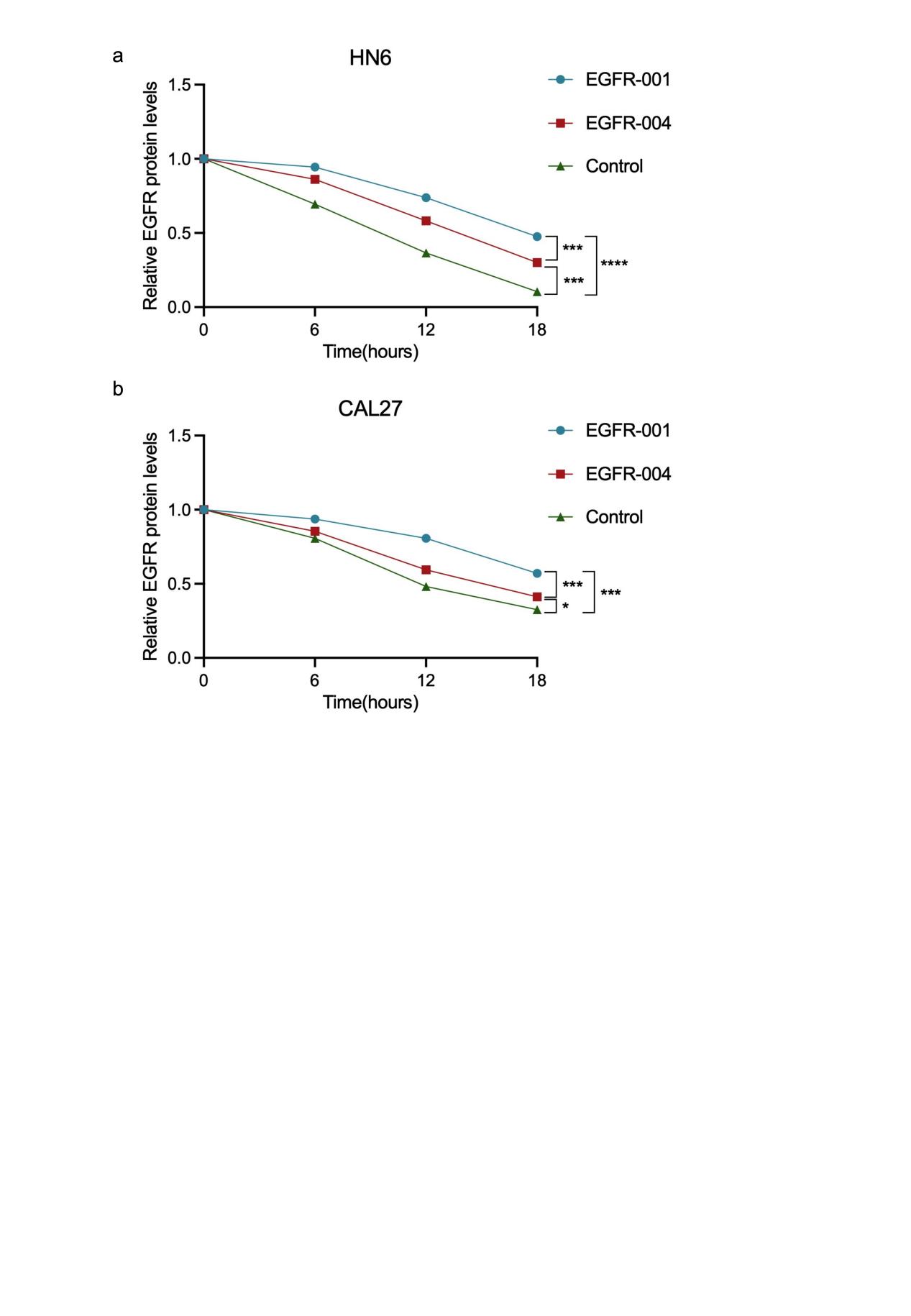


**Supplemental Figure 10.** The ABCA1 degradation rate was quantified by grayscale analysis.


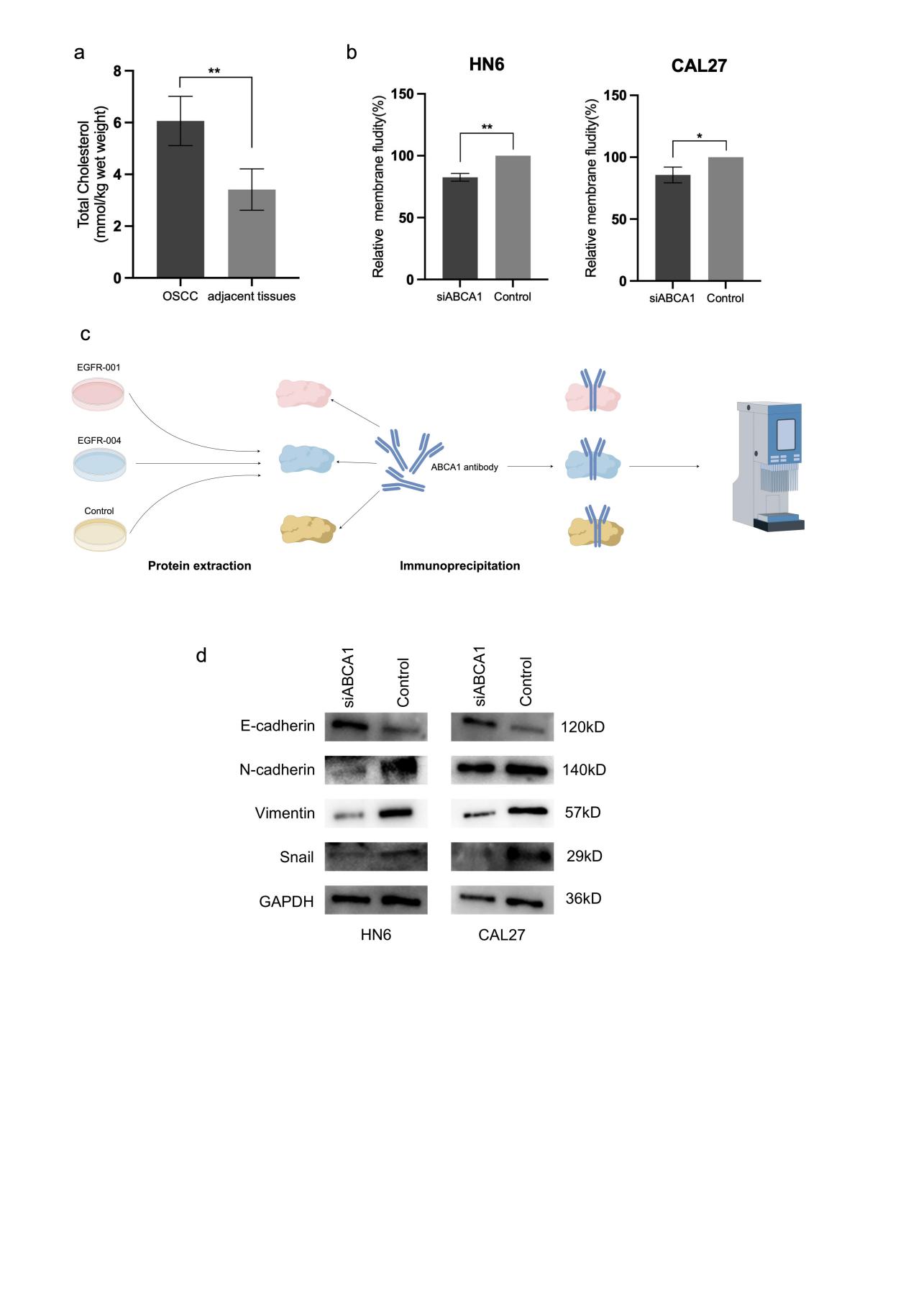


**Supplemental Figure 11.** ABCA1 modulated membrane fluidity and EMT through cholesterol efflux. (a) Total cholesterol contents of OSCC and adjacent tissues. (b) Effects of ABCA1 knockdown on cell membrane fluidity of HN6 and CAL27 cells. (c) The ABCA1 immunoprecipitation followed by proteomic screening by MS analysis. (d) Western blot assay detected the expression levels of EMT markers after ABCA1 was knocked down. Statistical significance was assessed with Student's t-test. * *P* < 0.05, ** *P* < 0.01.

**Supplemental Tables**

| **Table S1. Potential RBP in rs737540 sequence predicted by POSTAR3.** | | | | | | |
| --- | --- | --- | --- | --- | --- | --- |
| **RBP** | **Tissue type** | **Position** | **Method** | Scorea | **A1** | **A2** |
| TARDBP | HEK293T | chr7:55215647-55215676 | PAR-CLIP | 0.766 | T | C |
|  |  |  | PARalyzer |  |  |  |
| a PARalyzer score: T-to-C transition ratio, while higher ratio means more possible to bind RBPs. | | | | | | |

| **Table S2. The ABCA1 immunoprecipitation followed by proteomic screening**  **by MS analysis.** | | | | | |
| --- | --- | --- | --- | --- | --- |
| **Accession** | **Gene Name** | **Description** | **Unique Peptides** | | |
|  |  |  | **EGFR-001** | **EGFR-004** | **Control** |
| P08670 | VIM | Vimentin | 4 | 2 | 2 |
| P05783 | KRT18 | Keratin, type I cytoskeletal 18 | 5 | 3 | 1 |
| P06733 | ENO1 | Alpha-enolase | 1 | 1 | 1 |
| P04264 | KRT1 | Keratin, type II cytoskeletal 1 | 11 | 5 | 12 |
| P35908 | KRT2 | Keratin, type II cytoskeletal 2 | 4 | 1 | 5 |
| P35527 | KRT9 | Keratin, type I cytoskeletal 9 | 2 | 3 | 8 |
| P13645 | KRT10 | Keratin, type I cytoskeletal 10 | 3 | 1 | 5 |
| P08727 | KRT19 | Keratin, type I cytoskeletal 19 | 3 | 3 | 1 |

| **Table S3. Basic information of 67 samples included in the study.** | | |
| --- | --- | --- |
|  | Number（N = 67） | % |
| **Age** | | |
|  | 53.19 ± 12.09 |  |
| ≤50 | 31 | 46.27 |
| >50 | 36 | 53.73 |
| **Gender** | | |
| Male | 58 | 86.57 |
| Female | 9 | 13.43 |
| **Smoking** | | |
| non-smoking | 16 | 23.88 |
| ever smoking | 51 | 76.12 |
| **Drinking** | | |
| non-drinking | 30 | 44.78 |
| ever drinking | 37 | 55.22 |
| **TNM** | | |
| T | | |
| 1 | 7 | 10.45 |
| 2 | 35 | 52.24 |
| 3 | 13 | 19.4 |
| 4 | 10 | 14.83 |
| None | 2 | 2.99 |
| N | | |
| 0 | 42 | 62.69 |
| 1 | 12 | 17.91 |
| 2 | 11 | 16.42 |
| None | 2 | 2.99 |
| M | | |
| 0 | 65 | 97.01 |
| None | 2 | 2.99 |

| **Table S4. The sequences of primers and siRNAs that used for different experiments in this study.** | |
| --- | --- |
| **Product Name** | **Targeting Sequences** |
| EGFR-001 | F: GGAATACCTAAGGATAGCACCGC |
|  | R: CTGATGCATTTCATCTGAGCC |
| EGFR-004 | F: AGCAGCAGCCAGTCTCCAGT |
|  | R: TGCTCCTTACGCCCTTCACT |
| TARDBP | F: GGGAAATCTGGTGTATGTTGTCA |
|  | R: TTTTCTGGACTGCTCTTTTCACT |
| GAPDH | F: TGCTCCTTACGCCCTTCACT |
|  | R: GCCTGCTTCACCACCTTCT |
| ABCA1 | F: CAGAGGTGGCTCTGATGACC |
|  | R: TGTTTTGCTTTGCTGACCCG |
| E-cadherin | F: GCCTCCTGAAAAGAGAGTGGAAG R: TGGCAGTGTCTCTCCAAATCCG |
| N-cadherin | F: CCTCCAGAGTTTACTGCCATGAC  R: GTAGGATCTCCGCCACTGATTC |
| Snail | F: TGCCCTCAAGATGCACATCCGA  R: GGGACAGGAGAAGGGCTTCTC |
| Vimentin | F: AGAGGAGATGCAGGAGCTGA  R: GACTGCGGACAGACCTTCAA |
| siTARDBP | GGAGAGGACTTGATCATTA |
| siABCA1 | GGAGAUGUUACAAUAGUUUU |
| ASO(rs737540-T) | CAUGAAGAAGAAGGACAUUC |

| **Table S5. Sources of antibodies and the working dilutions that were used for different experiments in this study.** | | | |
| --- | --- | --- | --- |
| **Antibody** | **Vendor** | **Catalog no.** | **Uses** |
| Rabbit anti-ABCA1 | Affinity Biosciences (Cincinnati, OH, USA） | DF8233 | WB-1:2000; IP(2μg/reaction tube); IHC-1:50 |
|  |  |  |  |
|  |  |  |  |
| Rabbit anti-EGFR | Affinity Biosciences (Cincinnati, OH, USA） | AF6043 | WB-1:1000; IHC-1:50 |
| Rabbit anti-  pEGFRTyr1173 | Affinity Biosciences (Cincinnati, OH, USA） | AF3048 | WB-1:1000; IHC-1:50 |
| Rabbit anti-TDP-43 | Proteintech (Rosemont, IL, USA) | 10782-2-  AP | WB-1:2000 |
| Rabbit anti-GAPDH | Proteintech (Rosemont, IL, USA) | 80570-1-RR | WB-1:2000 |
| Rabbit anti-KI67 | Proteintech (Rosemont, IL, USA) | 27309-1-  AP | IHC-1:2000 |
| Rabbit anti-IgG | Proteintech (Rosemont, IL, USA) | 30000-0-  AP | IP(2μg/reaction tube) |
| Mouse anti-Ubiquitin | Cell Signaling Technology, Inc. (Beverly, MA) | #3936T | WB-1:1000 |
| Mouse anti-E-cadherin | Proteintech (Rosemont, IL, USA) | 60335-1-  IG | WB-1:2000; IHC-1:1000 |
| Mouse anti-N-cadherin | Proteintech (Rosemont, IL, USA) | 66219-1-  IG | WB-1:5000; IHC-1:7500 |
| Rabbit anti-Vimentin | Proteintech (Rosemont, IL, USA) | 80232-1-RR | WB-1:20000 |
| Rabbit anti-Snail | Cell Signaling Technology, Inc. (Beverly, MA) | #3879 | WB-1:1000 |
